# Sensory-guided Design of Garlic Using tea and Identification of key active components

**DOI:** 10.1016/j.fochx.2026.104379

**Published:** 2026-09-01

**Authors:** Yongxian Chen, Ying Xu, Haibo Zhang, Hong Huang, Ruixin Xue, Xiang Yuan, Xiangyang Guo, Pinwu Li

**Affiliations:** a Tea Resources Utilization and Quality Testing Key Laboratory of Sichuan Province, College of Horticulture, Sichuan Agricultural University, Chengdu 611130, Sichuan, PR China; b College of Tea and Food Science, Xinyang Normal University, Xinyang 464000, China

**Keywords:** Tea, Garlic, Tea polyphenols, Volatile components

## Abstract

Garlic is widely used as a condiment, yet its sulfur-derived odor persists long after consumption, often limiting sensory acceptance. Using common dietary components to modulate garlic odor is viewed as a promising sensory-guided design strategy for garlic. In this study, we compared the odor characteristics and volatile compositions of original garlic paste, distilled water-treated garlic paste, and tea aqueous extract-treated garlic paste through sensory evaluation and chemical analysis. The results demonstrated that tea treatment significantly inhibited the formation of 12 key sulfur-containing compounds, including diallyl disulfide and thiophene derivatives, resulting in a 37.10% reduction in total sulfur-containing volatiles. Using diallyl disulfide as a representative odor-active compound and its clearance rate as an indicator, tea polyphenols were identified as the primary contributors to odor modulation. Rather than simply eliminating odor, tea-mediated interactions provided a chemical basis for the sensory-guided design of garlic odor characteristics.

## Highlights

• Tea treatment reduced sulfur-derived volatiles in garlic by 37.10%.
• Twelve key sulfur odorants, including diallyl disulfide, were selectively inhibited.
• Tea polyphenols drive sensory-guided chemical modulation of garlic aroma.

## Graphical abstract

**Figure:**
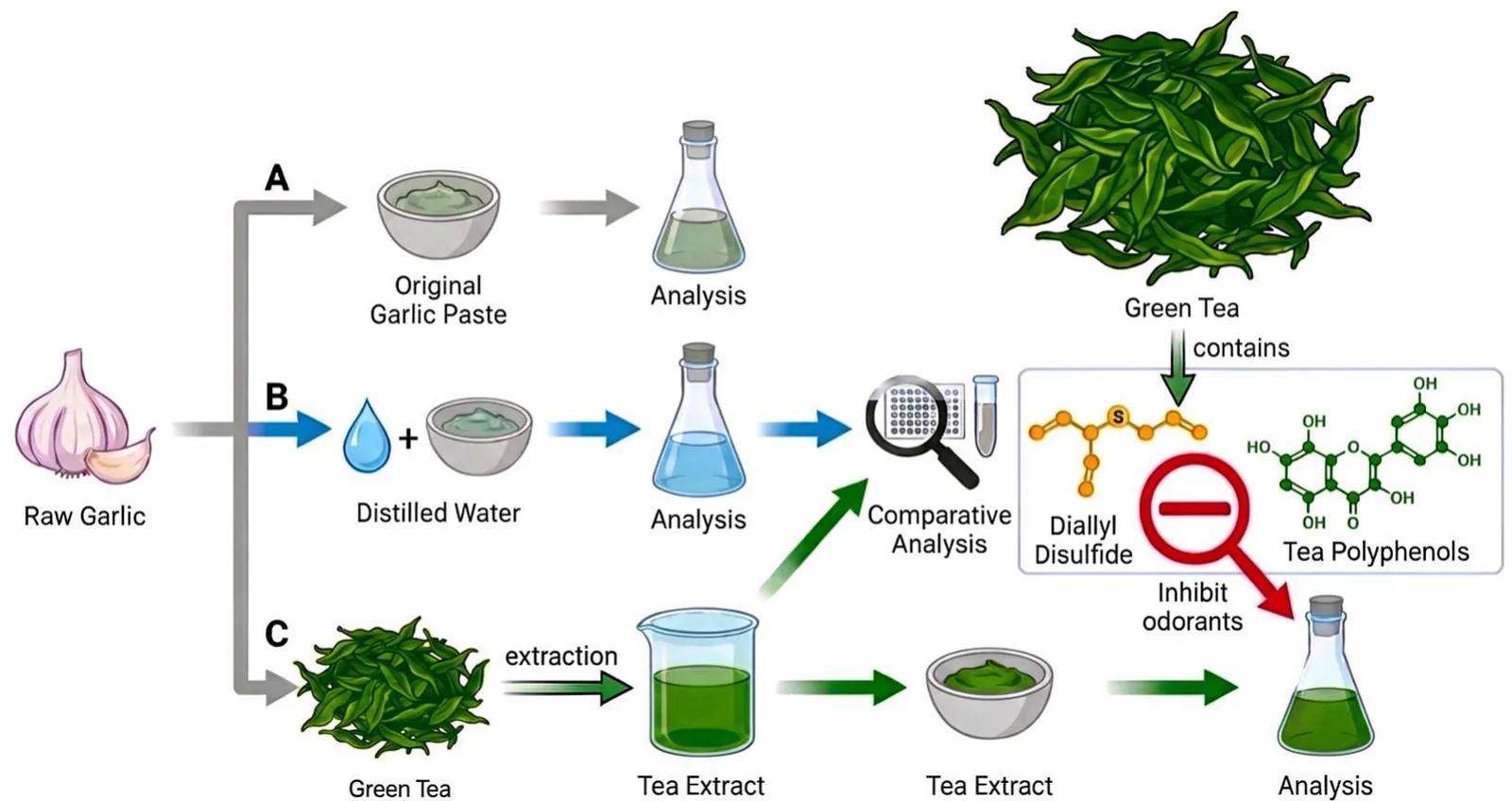

## 1 Introduction

Garlic (*Allium sativum*) is widely consumed and highly regarded by consumers for its distinctive aroma, contributing to enhanced flavor and complexity of foods. In addition to its culinary value, garlic provides multiple health benefits, including antibacterial, anti-inflammatory, and antioxidant properties (Huang et al., 2022; Mousa et al., 2022; Nadeem et al., 2022). The characteristic garlic aroma primarily arises from tissue disruption, which triggers the enzymatic conversion of odorless sulfur-containing precursors into allicin and other sulfur-derived volatiles, resulting in a strong garlic flavor (Abe et al., 2021). Allicin can spontaneously decompose into other sulfur-containing compounds at room temperature, which can then volatilize into the air (Borlinghaus et al., 2021). Previous studies have shown that volatile compounds in fresh garlic are dominated by sulfur-containing compounds, accounting for approximately 75.78% of the overall volatile profile, with diallyl disulfide being the most abundant odor-active component responsible for pungent and characteristic garlic aroma (Wang et al., 2021). Following garlic tissue disruption, sulfur-containing volatile compounds are rapidly released, generating the characteristic garlic odor. Among these compounds, fat-soluble sulfur compounds, such as allicin and diallyl disulfide, are the primary contributors to the pungent aroma, whereas water-soluble sulfur-containing compounds are largely non-pungent. Notably, these sulfur-containing compounds are key to garlic's characteristic aroma. However, these properties also make garlic odor difficult to modulate in a mild and food-compatible manner without disrupting its overall flavor profile.

To address this challenge, numerous studies have investigated strategies to modulate garlic odor, employing approaches such as β-cyclodextrin inclusion, secondary encapsulation of garlic powder using fluidized-bed technology, and microwave treatment. Although these methods can attenuate odor intensity, their application is often limited by processing complexity, high cost, and unintended alterations to garlic's original flavor profile. These limitations highlight the need for alternative strategies for modulating garlic aroma while preserving its inherent sensory attributes (Guan et al., 2018; Qi et al., 2004). In this context, food-based sensory design strategies that rely on naturally occurring dietary components offer a promising alternative. Tea, as a widely consumed beverage and a rich source of bioactive compounds, represents a suitable candidate for such food-based approaches (Li & Zhang, 2017).

Tea is rich in diverse bioactive components, such as tea polyphenols, theanine, caffeine, and polysaccharides, with tea polyphenols standing out for their pronounced chemical reactivity relevant to sensory modulation (Huang et al., 2022; Yang et al., 2013). Tea has been reported to interact with sulfur-containing volatile compounds through chemical mechanisms rather than simple physical masking. In particular, tea polyphenols exhibit strong chemical reactivity that enables modulation of sulfur-containing volatiles, which are responsible for the characteristic and persistent odor of garlic (Bi et al., 2023). Tea polyphenols, particularly flavonoids and catechins, are characterized by multiple phenolic hydroxyl groups that confer strong chemical reactivity (Yao et al., 2025). This intrinsic reactivity enables interactions with volatile odor-active compounds, providing a molecular basis for modulating sulfur-derived odor characteristics in foods (Ma & Huang, 2014; Yu et al., 2023). Previous studies have demonstrated that tea-derived materials can interact with odor-active volatile compounds, supporting the potential of tea constituents for modulating undesirable odor characteristics (Takahashi et al., 2011). Importantly, tea polyphenols have also been shown to maintain such interactions within complex matrices, highlighting their robustness and suitability for odor modulation in food-related systems (Jiang et al., 2013). Such interactions are particularly pronounced for odor-active molecules containing reactive sulfur and nitrogen functional groups, indicating that chemical reactivity may underlie the odor-modulating effects of tea-derived components (Gong & Tang, 2000). However, it remains unclear how these interactions specifically affect the profile of sulfur-containing volatiles in garlic. Moreover, whether such chemically driven interactions translate into perceptible changes in garlic aroma has not been systematically evaluated. From a food-based sensory design perspective, elucidating the interaction between tea components and sulfur-derived garlic odor is necessary to understand how garlic aroma can be modulated while preserving its intrinsic sensory properties.

Although tea and its polyphenolic constituents can interact with sulfur-containing odor-active compounds, their application as a sensory design tool for garlic remains unclear. In particular, how chemically driven interactions induced by tea treatment contribute to the rational design and reshaping of garlic aroma at both the chemical and sensory levels has not been comprehensively elucidated. Therefore, this study integrates instrumental analysis of volatile compounds with sensory evaluation. This approach establishes a multimodal, sensory-guided framework linking chemical changes in sulfur-derived volatiles with corresponding sensory responses. It further allows us to investigate the role of tea and its key active components in modulating garlic odor. By providing mechanistic insight into tea-guided garlic flavor design, this work aims to advance the understanding of food-based sensory design and reformulation strategies using naturally derived dietary components.

## 2 Materials and methods

### 2.1 Materials

Purple-skinned garlic (*Allium sativum* L.), green tea (Mengding Ganlu Grade I), and dark tea (Ya'an Tibetan Tea Grade II) were selected as the primary plant materials. Tetradecane (Shanghai Yuanye Biotechnology Co., Ltd., Shanghai, China); (*R*)-(−)-^2^H_4_-carvone (Yunnan Xili Biotechnology Co., Ltd., Yunnan, China) and diallyl disulfide (DADS) standard (Nanjing Yuanzhi Bio-technology Co., Ltd., Nanjing, China) were purchased for analytical purposes.

### 2.2 Instrument

Multi-functional pulverizer (Baijie BJ-150); electronic balance (Sartorius BCE224i-1CCN); electrothermal thermostatic water bath (Tester SYG-2-8); high-speed refrigerated centrifuge (Thermo Fisher ST 16R); gas chromatography (Agilent 7890 A-5975C); CNC ultrasonic cleaner (Kunshan Wo Chuang KH5200DE); rotary evaporator (Shanghai Xian De xiande-2000 A); UV–visible spectrophotometer (Shanghai Youke UV759); circulating water vacuum pump (Yuhua Instruments SHZ-DIII); High-performance vacuum pump (Yuhua Instruments); high-performance liquid chromatograph (Agilent 1260 Infinity II).

### 2.3 Experimental methods

#### 2.3.1 Sensory evaluation of the effect of tea on garlic odor

The dried tea samples were powdered and passed through a 40-mesh sieve. Then, 1 g of tea powder was extracted with 50 mL of distilled water in boiling water for 30 min. The material-to-liquid ratio was 1:50 (*w*/*v*). After centrifugation, the supernatant was collected as the tea aqueous extract. For the garlic treatment, 1 g of garlic paste was used for each sample. For the water-treated samples, 1 g of garlic paste was mixed with 5 mL of distilled water for 5 min. For the tea-treated samples, 1 g of garlic paste was mixed with 5 mL of tea aqueous extract for 5 min. After treatment, the mixture was filtered. The filtrate was discarded, and the treated garlic paste was collected for further analysis.

A group of ten individuals with normal olfactory ability was recruited to evaluate the odor of untreated and treated garlic paste. Sensory scores were obtained according to predefined criteria, and the mean of highest and lowest scores was calculated for each treatment. For each replicate, panelists and sample order were randomly assigned by drawing lots, and evaluation criteria are shown in Table S1. No personally identifiable information was collected, and all data were recorded and analyzed anonymously.

#### 2.3.2 Detection of volatile components of garlic paste by GC–MS

The volatile profile of garlic paste, following its treatment with a tea aqueous extract, was analyzed using headspace solid-phase microextraction coupled with gas chromatography–mass spectrometry (HS-SPME-GC–MS), with tetradecane serving as the internal standard. This analysis was used to compare volatile compounds among untreated garlic paste, distilled water-treated garlic paste, and tea aqueous extract-treated garlic paste, with a focus on sulfur-containing volatiles. The GC–MS detection conditions were based on a previous method (Wang et al., 2021). The chromatographic column was Agilent 19091S-433 HP-5MS (30 m × 0.25 mm × 0.25 μm). Characterization and quantification methods: characterization was performed by NIST 14 mass spectrometry database and references, and quantification was performed based on the peak area of the internal standard and the peak area of each component of the sample at known mass concentration with the following formula:

(1) $$\text{Target substance content}\left(\mathrm{\mu g}/g\right)=\frac{A_{x}\times \rho_{i}\times V_{i}}{A_{i}\times m}$$

In this equation, $A_{x}$ denotes the peak area of the target, $A_{i}$ represents the peak area of the internal standard, $\rho_{i}$ signifies the mass concentration of the internal standard that has been added, with a value of 76.28 μg/mL, $V_{i}$ represents the volume of the internal standard that has been added, with a value of 0.01 mL, and m represents the mass of the sample in grams.

#### 2.3.3 Determination of volatile components of garlic tea mixture by GC–MS

The volatile compounds in the sample were analyzed using fully automated headspace solid-phase microextraction (HS-SPME) coupled with gas chromatography–mass spectrometry (GC–MS), with (*R*)-(−)-^2^H_4_-carvone as the internal standard. This analysis was used to characterize a broader range of aroma compounds in the garlic–tea mixture for sensory flavor analysis. The results from the two GC–MS analyses were analyzed separately and were not directly compared.

The column was an Agilent J&W DB-5MS capillary column (30 m × 0.25 mm × 0.25 μm). The qualitative and quantitative analysis was performed as follows. Compound identification was based on a self-built database, published literature, reference standards, and retention indices. For selected ion monitoring, one quantitative ion and two to three qualitative ions were selected for each compound. The target ions were monitored in different time windows according to the elution order of the peaks. A compound was identified when its retention time matched that of the reference standard and the selected ions were present in the mass spectrum after background subtraction. The quantitative ions were used for peak integration and correction to improve the accuracy of quantification. The content of the target substance was calculated according to the following formula:

(2) $$\text{Target substance content}\left(\mathrm{\mu g}/g\right)=\frac{A_{x}\times \rho_{i}\times V_{i}}{A_{i}\times V}$$

In this equation, $A_{x}$ denotes the peak area of the target, $A_{i}$ represents the peak area of the internal standard, $\rho_{i}$ signifies the mass concentration of the internal standard that has been added, with a value of 50 μg/mL, $V_{i}$ represents the volume of the internal standard that has been added, with a value of 0.01 mL, and V represents the volume of the sample in mL.

#### 2.3.4 Calculation of DADS clearance rate

The characterization and quantification of DADS were carried out using standards. The peak area of DADS standard solution was detected by high performance liquid chromatography (HPLC), and the standard curve was made with DADS concentration as the horizontal coordinate and DADS peak area as the vertical coordinate.

The extraction of DADS from homogenized garlic was performed with methanol. The mixture was centrifuged, and the supernatant was filtered through a 0.45 μm membrane. DADS content in the extract was quantified by HPLC, and the DADS clearance rate was calculated according to the following formula:

(3) $$\text{DADS clearance}\left(\%\right)=\frac{\rho_{0}-\rho_{1}}{\rho_{0}}\times 100\%$$

In this equation, $\rho_{0}$ represents the DADS concentration of the blank control and $\rho_{1}$ represents the DADS concentration of the sample treatment, both in μg/mL. HPLC analysis was performed according to the method described by Qu (2010), with modifications. The separation was achieved using an Agilent ZORBAX SB-C18 column (250 mm × 4.6 mm, 5 μm) maintained at 25 °C. The mobile phase consisted of methanol and water (70:30, *v*/v) delivered at a flow rate of 0.8 mL/min. The detection was carried out with a UV detector set at 240 nm, and the injection volume was 15 μL.

#### 2.3.5 Test on the removal effect of tea polysaccharides and tea saponins

Tea polysaccharide removal treatment was performed according to a previously reported method (Huang et al., 2012). Tea polysaccharides were extracted from the tea powder with distilled water using ultrasonic and boiling water bath treatments. The polysaccharides were then precipitated from the supernatant by adding two volumes of anhydrous ethanol, and the resulting filtrate was collected as the tea polysaccharide-removed sample. Tea saponin removal was conducted according to a previously reported method (Yu et al., 2015). Tea saponin was extracted from tea powder using 95% ethanol with ultrasonic and thermal assistance. The saponins were then precipitated from the supernatant by adding two volumes of acetone, and the resulting filtrate was collected as the tea saponin-removed sample. The supernatant and filtrate were each mixed with an equal volume of DADS extract and allowed to react for 5 min. A blank control, prepared in the same manner but without tea powder, was run concurrently. The DADS clearance rate for both the supernatant and filtrate was then calculated.

#### 2.3.6 DADS clearance test of different tea fractions

The extraction method was based on a previous reference (Lü et al., 2013). Aqueous extracts were obtained from tea powder through repeated extraction in a boiling-water bath followed by concentration. The resulting solution was fractionated stepwise using organic solvents. After solvent removal, each fraction was reconstituted and analyzed for the content of tea polyphenols, caffeine, amino acids, and pigments. The DADS clearance capacity of each fraction was then evaluated by mixing it with DADS extract.

#### 2.3.7 Screening of tea characteristic components

Based on the primary polynomial regression model, multiple linear regression analysis was performed to evaluate the effect of characteristic tea components on the DADS clearance rate. The contents of tea polyphenols, caffeine, free amino acids, theaflavin, thearubigins, and theabrownins were used as independent variables. The DADS clearance rate was used as the dependent variable. This analysis was used to identify the characteristic tea components that had a significant effect on DADS clearance.

The crude tea polyphenol fraction was self-prepared in this study. Briefly, the aqueous fraction obtained after ether extraction was concentrated by rotary evaporation and then redissolved. The crude tea polyphenol solution was further separated using an AB-8 macroporous resin column with a stepwise methanol gradient from 0% to 100%. Each eluted fraction was assessed for its DADS clearance capacity. Finally, the tea polyphenol and catechin contents in each eluate fraction were quantified. The non-catechin fraction was calculated by subtracting the total catechin content from the total tea polyphenol content. This allowed us to assess the specific contribution of different polyphenolic fractions to the overall scavenging effect.

#### 2.3.8 Determination methods of tea components

The contents of tea polyphenols and catechins were determined using the Folin-Ciocalteu method and high-performance liquid chromatography (HPLC), respectively, in accordance with the Chinese National Standard GB/T 8313–2018. Caffeine content was analyzed by UV–Vis spectrophotometry following GB/T 8312–2013. Free amino acids were determined by the ninhydrin method according to GB/T 8314–2013. Theaflavin, thearubigin, and theabrownin were determined by the systematic analysis method according to Roberts and Smith (1961).

#### 2.3.9 Statistical analysis

Statistical analyses were performed using Excel 2019, SPSS 27, and Origin 2024. All experiments were conducted in triplicate, and data are expressed as mean ± standard deviation. Group differences were assessed by Duncan's multiple range test. A probability value of *p* < 0.05 was considered statistically significant, and different superscript letters were used to denote significant differences in the results.

## 3 Results and discussion

### 3.1 Sensory evaluation results

Sensory evaluation was conducted according to the garlic odor intensity criteria listed in Table S1. A higher score indicated a weaker garlic odor and better odor acceptability. As shown in Table 1, the untreated garlic paste received the lowest score of 1.68, corresponding to a strong pungent garlic odor. Distilled water treatment increased the score to 5.71, indicating that the garlic odor was reduced to a moderate level. The green tea aqueous extract treatment obtained the highest score of 8.41, corresponding to a negligible garlic odor. In addition, this sample exhibited a mild tea aroma, which made the overall odor more acceptable. These results suggest that green tea aqueous extract improved garlic odor not only by reducing the pungent odor intensity, but also by introducing favorable aroma notes and improving the overall odor profile.

**Table 1:** Sensory evaluation scores of garlic paste samples under different treatments.

Treatment	Garlic Odor Intensity	Score
Control	Intense pungent garlic odor	1.68 ± 0.06
Distilled water	Moderate garlic odor	5.71 ± 0.09
Tea aqueous extract	Negligible garlic odor	8.41 ± 0.07

### 3.2 Results of GC–MS detection of volatiles from garlic paste with different treatments

Since distilled water also reduced garlic odor in the sensory evaluation, the volatile compounds in three garlic paste samples, including the untreated control, distilled water treatment, and tea aqueous extract treatment, were analyzed by GC–MS (Table S2). In total, 36 compounds were identified: 17 thioethers, 16 heterocyclics, 2 thiols, and 1 non–sulfur-containing compound. 2-Thiophenemethyl mercaptan and allyl mercaptan were absent in the untreated group, whereas propylene sulfide was not detected in the distilled water treatment group. In the tea aqueous extract treatment group, propylene sulfide, 3-methyl-1,2-dithia-3-cyclopentene, and 2-thiophenemethyl mercaptan were not detected. Among all treatments, DADS exhibited the highest content.

**Fig. 1:**
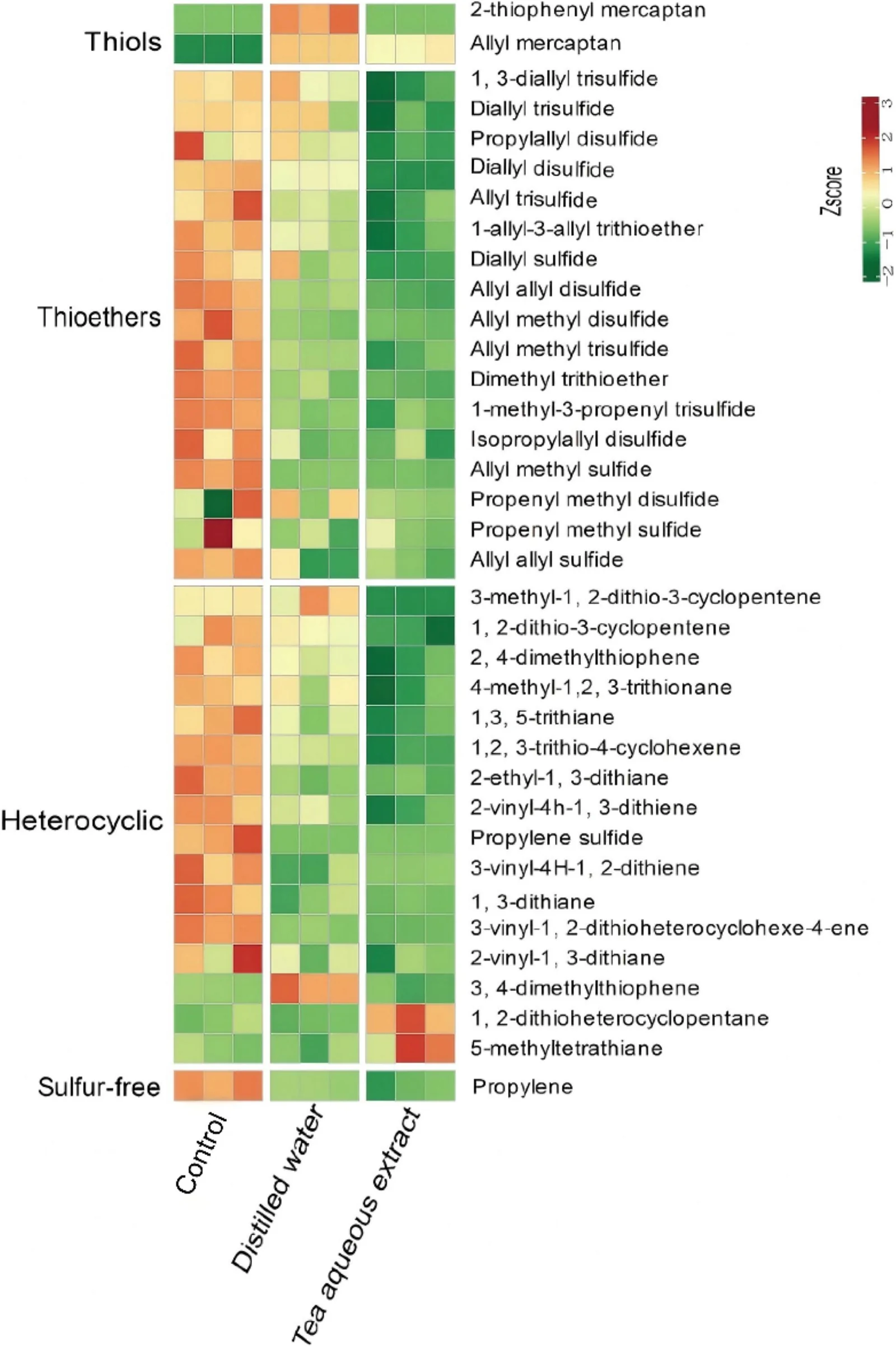
Heatmap of major volatile compounds in garlic paste samples under different treatments. Control, untreated garlic paste; Distilled water, garlic paste treated with distilled water; Tea aqueous extract, garlic paste treated with green tea aqueous extract. (For interpretation of the references to colour in this figure legend, the reader is referred to the web version of this article.)

As shown in Fig. 1, the heatmap clearly showed differences in the major volatile compounds among the untreated control, distilled water-treated, and tea aqueous extract-treated garlic paste samples. Compared with the untreated control, both distilled water and tea aqueous extract treatments reduced the contents of many sulfur-containing volatiles. The tea aqueous extract-treated sample showed lower levels of most pungent sulfur-containing compounds than the distilled water-treated sample, indicating a stronger deodorizing effect. A few compounds showed different changes among the three treatments. Overall, these results indicate that distilled water contributed to a partial reduction of garlic odor volatiles, while tea aqueous extract showed a stronger effect, supporting the role of tea constituents in reducing sulfur-derived garlic odor.

The findings confirm that the aqueous solvent (distilled water) itself contributed to a measurable reduction in garlic odor volatiles. However, the tea aqueous extract demonstrated a superior deodorizing efficacy, achieving a significantly greater reduction. This enhanced effect, consistent with sensory evaluation data, underscores that the role of tea constituents extends beyond mere solvent effects.

**Fig. 2:**
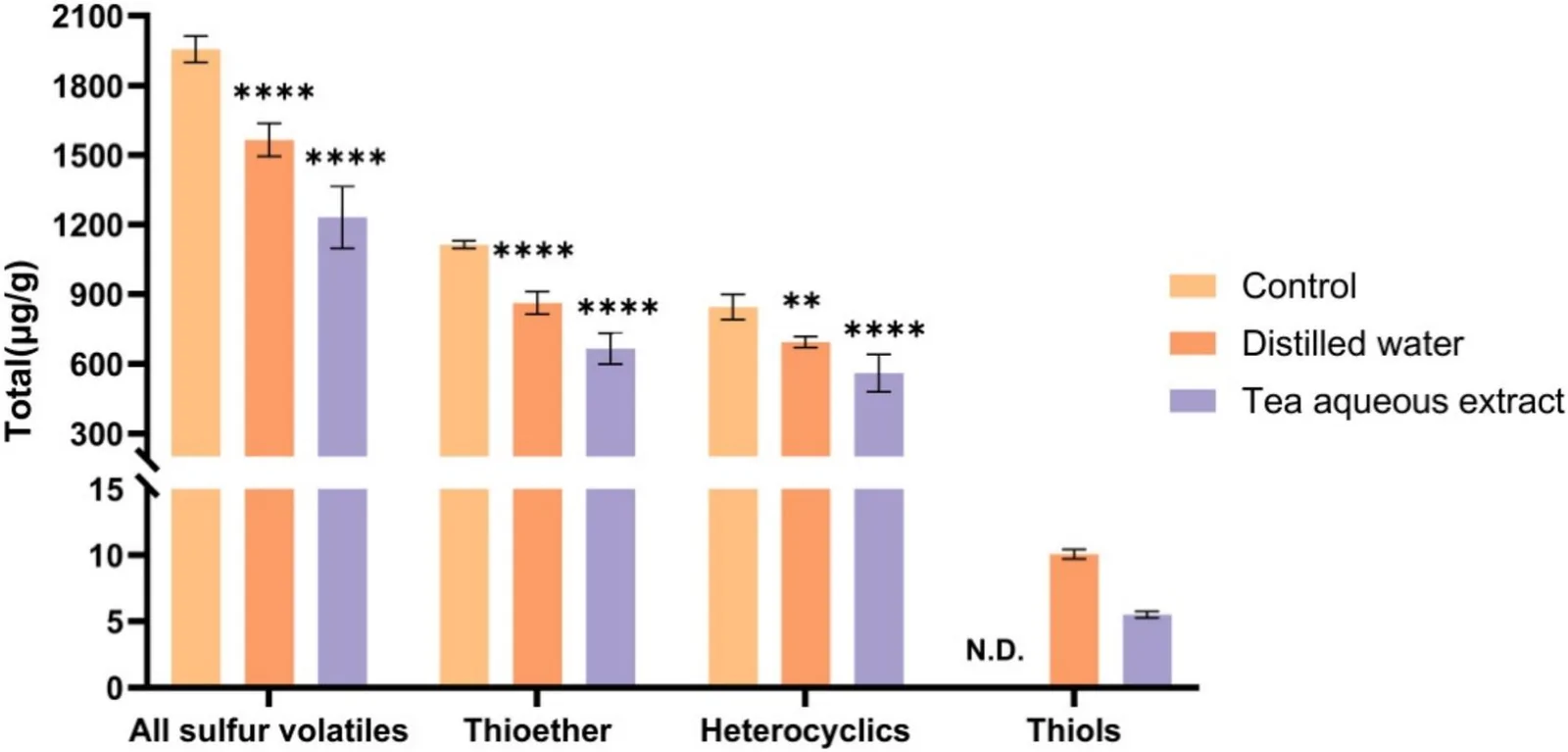
Total amount of volatile compounds in each category under different treatments.

The calculated total amounts are shown in Fig. 2. The total sulfur-containing volatiles in the original garlic paste amounted to 1955.58 μg/g. Compared to the untreated sample, the distilled water treatment reduced this by 19.99%, while the tea aqueous extract treatment reduced it by 37.10%. The total sulfur-containing volatiles in the distilled water-treated and tea aqueous extract-treated samples were 1564.75 μg/g and 1230.11 μg/g, respectively. The total sulfur ether content was 1112.01 μg/g for the untreated sample, 861.61 μg/g for the distilled water-treated sample, and 665.13 μg/g for the tea aqueous extract-treated sample. The total heterocyclic compounds was 843.57 μg/g in the untreated sample, 693.06 μg/g in the distilled water-treated sample, and 559.48 μg/g in the tea aqueous extract-treated sample. The total thiols was 10.07 μg/g in the distilled water-treated sample and 5.50 μg/g in the tea aqueous extract-treated sample, while the untreated sample contained none. Distilled water reduced the overall abundance of sulfur-containing volatiles.

Notably, however, it increased the thiol content compared within the original garlic paste. In contrast, the tea aqueous extract not only replicated this general reduction but also further reduced the levels of all individual species categories, including thiols.

For the representative sulfur-containing compounds in garlic, including diallyl sulfide, diallyl disulfide and diallyl trisulfide, the odor activity value (OAV) was used as an index to evaluate their sensory impact, calculated as the concentration of each volatile compound divided by its corresponding odor threshold. The calculated OAVs are shown in Table 2. The OAV of diallyl disulfide was much higher than those of diallyl sulfide and diallyl trisulfide, indicating that diallyl disulfide made a greater contribution to the pungent odor of garlic. After treatment with tea aqueous extract, the OAV of diallyl disulfide was almost half of that in the untreated garlic paste. This reduction may explain the clear sensory difference after tea treatment. Therefore, diallyl disulfide was selected as the representative odor-active compound for evaluating garlic odor reduction.

**Table 2:** Odor activity values of three thioethers.

Compounds	Thresholds (mg/kg)	Untreated control (OAV)	Distilled water treatment (OAV)	Tea aqueous extract treatment (OAV)
Diallyl sulfide	0.30	309.55	290.04	268.84
Diallyl disulfide	0.08	6200.48	4999.16	3260.49
Diallyl trisulfide	0.01	4167.03	4120.98	3091.85

Note: OAV is dimensionless.

**Fig. 3:**
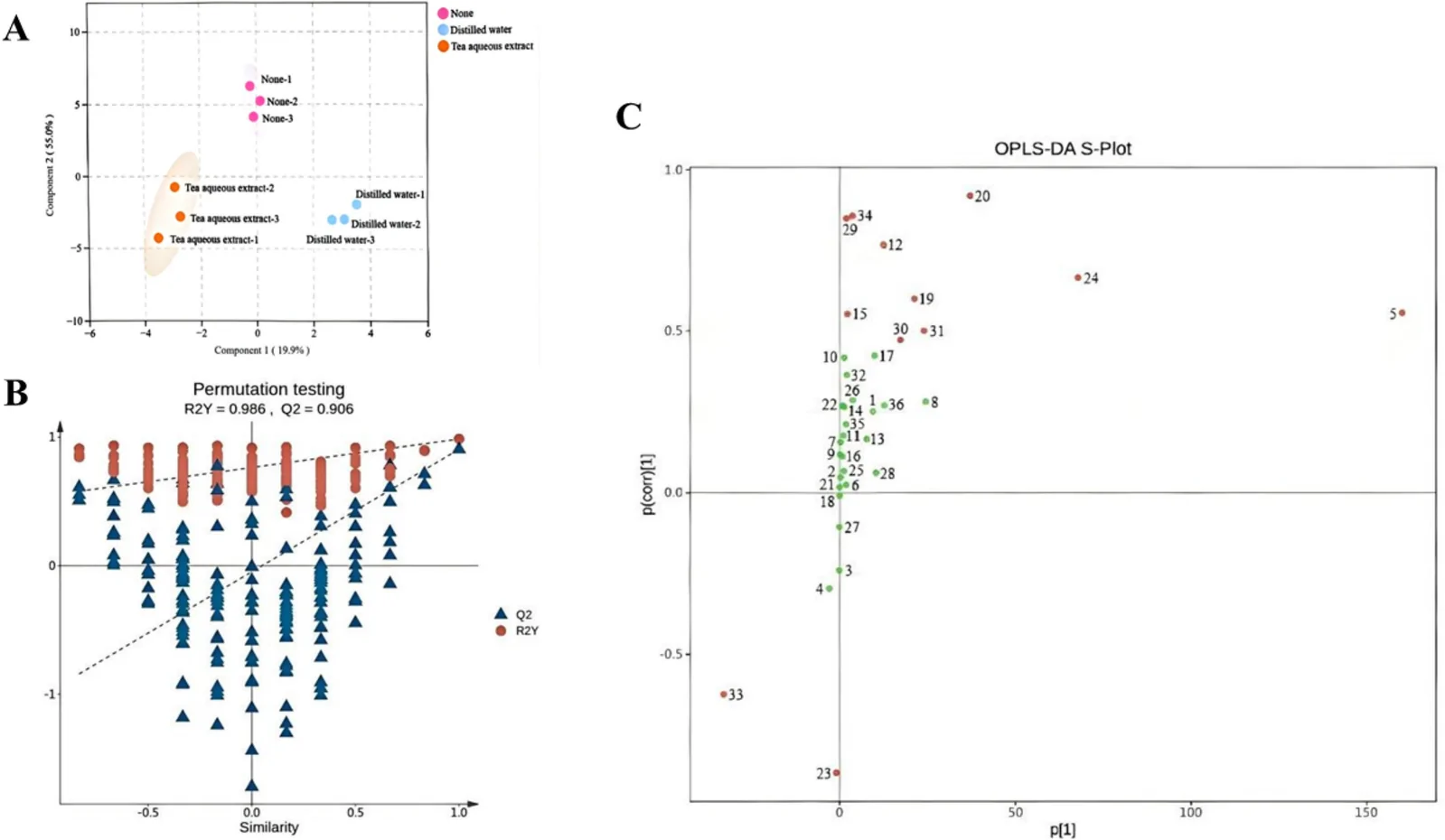
OPLS-DA analysis and identification of differential volatile compounds in garlic paste under different treatments. (A) OPLS-DA score plot of volatile components. (B) Permutation test of the OPLS-DA model. (C) OPLS-DA S-plot showing differential volatile compounds.

Fig. 3A and B show the OPLS-DA score plot and permutation test plots of volatile components for the untreated control, distilled water treatment, and tea aqueous extract treatment. The untreated control was distributed near the boundary between the first and second quadrants. The distilled water treatment was distributed in the fourth quadrant, while the tea aqueous extract treatment was distributed in the third quadrant. The three treatment groups were clearly separated, indicating significant differences in volatile components among the different treatments. For the OPLS-DA model, parameters closer to 1 indicate better model stability and reliability. Q^2^ indicates the predictive ability of the model. A model is generally considered valid when Q^2^ is greater than 0.5 and excellent when Q^2^ is greater than 0.9. The model had R^2^Y = 0.986 and Q^2^ = 0.906, indicating a good fit. The Q^2^ regression line crossed the horizontal axis, and its intercept with the vertical axis was negative (0.0, −0.047). This indicates that the statistical model was valid and showed no overfitting. Normally, the model is considered significant when the *p*-value in the cross-validation analysis is less than 0.05, and the *p*-value of this model was 0.005, indicating that the OPLS-DA model developed by the experiment is statistically significant.

In Fig. 3C, the horizontal coordinates indicate the covariance between the principal components and the substances, and the vertical coordinates indicate the correlation coefficient between the principal components and the substances. The closer a point is to the upper-right or lower-left corner, the more significant the difference is. The VIP value is the weight value of the variables in the OPLS-DA model. It indicates the intensity of the influence of the intergroup differences of the corresponding substances in the classification discrimination of each group of samples in the model, and the larger the value of VIP is, the larger is the contribution. A substance with a VIP value greater than 1 is considered to be significantly different. The green dots have a VIP value less than or equal to 1 and there are 24 of them. The red dots have VIP values greater than 1. In descending order, they are 3,4-dimethylthiophene, 1,2-dithiolane, 2-thiophenemethylthiol, 3-methyl-1,2-dithia-3-cyclopentene, diallyl trisulfide, 1,2-dithia-3-cyclopentene, 5-methyltetrathiazane, 2,4-dimethylthiophene, diallyl disulfide, 1,3-dipropenyl trisulfide, 1,2,3-trithia-4-cyclohexene, and 4-methyl-1,2,3-trithia cyclopentane, which are 12 substances that are differential volatiles between the three treatments.

### 3.3 Sensory flavor analysis

Twenty-five of the 36 substances detected in all treated garlic pastes had odor profiles retrieved according to references and public flavor databases, as shown in Table 3 (Chen et al., 2018; Fu et al., 2021; Zhen et al., 2020). Sulfur ethers were more abundant than the other categories. Among them, diallyl sulfide (No. 1), allylmethyl disulfide (No. 6), and dimethyl trisulfide (No. 11) each had seven odor descriptors, while diallyl disulfide (No. 5) and diallyl trisulfide (No. 12) each had six odor descriptors. These compounds are important sources of garlic flavor.

**Table 3:** Odor description of garlic volatile compounds.

Number	Compounds	Odor threshold (mg/kg)	Odor£
1	Diallyl sulfide	0.3	Garlic aroma，petroleum-like odor，Sulfurous odor，Onion odor，Garlic odor，Pungent horseradish odor，Metallic odor
2	Allyl methyl sulfide	0.022	Pungent garlic odor，Pungent garlic flavor，Garlic odor，Onion odor
4	Allyl propenyl sulfide	NA	Irritating odor，Burnt odor，Pungent garlic flavor
5	Diallyl disulfide	0.08	Onion odor，Garlic odor，Pungent garlic flavor，Garlic aroma，Pungent mustard aroma，Irritating odor
6	Allyl methyl disulfide	0.035	Onion odor，Garlic aroma，Chive-like odor，Scallion aroma，Garlic odor，Fresh scent，Leafy aroma
7	Propenyl methyl disulfide	0.0063	Delicate fragrance，Pungent garlic odor，Scallion aroma
8	Allyl propenyl disulfide	NA	Pungent garlic flavor
9	Isopropyl allyl disulfide	NA	Spicy odor，Chive-like odor
11	Dimethyl trisulfide	0.006	Chive-like odor，Fishy odor，Pungent garlic flavor，Cured meat aroma，Sulfurous odor，Cooked aroma，Onion odor
12	Diallyl trisulfide	0.01	Garlic aroma，Irritating odor，Sulfurous odor，Pungent garlic flavor，Garlic odor，Scallion aroma
13	Allyl methyl trisulfide	0.021	Mushroom odor，Pungent garlic odor，Scallion aroma，Onion odor，Creamy aroma
14	Propyl allyl trisulfide	NA	Garlic odor，Onion odor
18	Propylene sulfide	NA	Irritating odor，Spicy odor
19	2,4-Dimethylthiophene	3.00	Green
20	3,4-Dimethylthiophene	5.00	Charcoal-grilled aroma，Onion odor，Pungent garlic flavor
21	1,3-Dithiane	NA	Garlic aroma，Charcoal-grilled aroma
24	1,2-Dithiacyclopentene	NA	Pungent garlic flavor
25	2-Vinyl-1,3-dithiane	NA	Garlic aroma，Onion odor，Charcoal-grilled aroma
26	2-Vinyl-4H-1,3-dithiin	NA	Pungent garlic flavor
27	3-Vinyl-4H-1,2-dithiin	NA	Pungent garlic flavor，Irritating odor
28	3-Vinyl-1,2-dithiacyclohex-4-ene	N A	Green，Pungent garlic flavor
29	3-Methyl-1,2-dithiacyclopent-3-ene	NA	Mushroom odor
32	1,3,5-Trithiane	NA	Garlic aroma
34	Thiophen-2-ylmethanethiol	NA	Baked aroma，Coffee aroma，Fishy odor
35	Allyl mercaptan	NA	Pungent garlic flavor，Garlic odor，Sulfurous odor，Irritating odor

£ Note: Odor thresholds were obtained from published literature (Sun et al., 2025; Xie et al., 2022). NA, not available.

In addition, a search of the GC–MS results for the garlic tea mixture identified 126 aroma-related compounds, listed in Table S3. The odor descriptors of these substances primarily indicated positive attributes, contributing to masking and improving the garlic odor. Among them, trans-2,4-decadienal (No. 42) was a characteristic aroma component of dark tea, geranyl formate (No. 87) had the odor of tea, leaf alcohol (No. 115) also had the odor of new tea leaves, and cis-3-hexenol benzoate (No. 158) was the main aroma component of jasmine tea. It accounted for about 25% of the total aroma components in jasmine tea, while it was present at very low levels in green, black, oolong, and post-fermented teas.

**Fig. 4:**
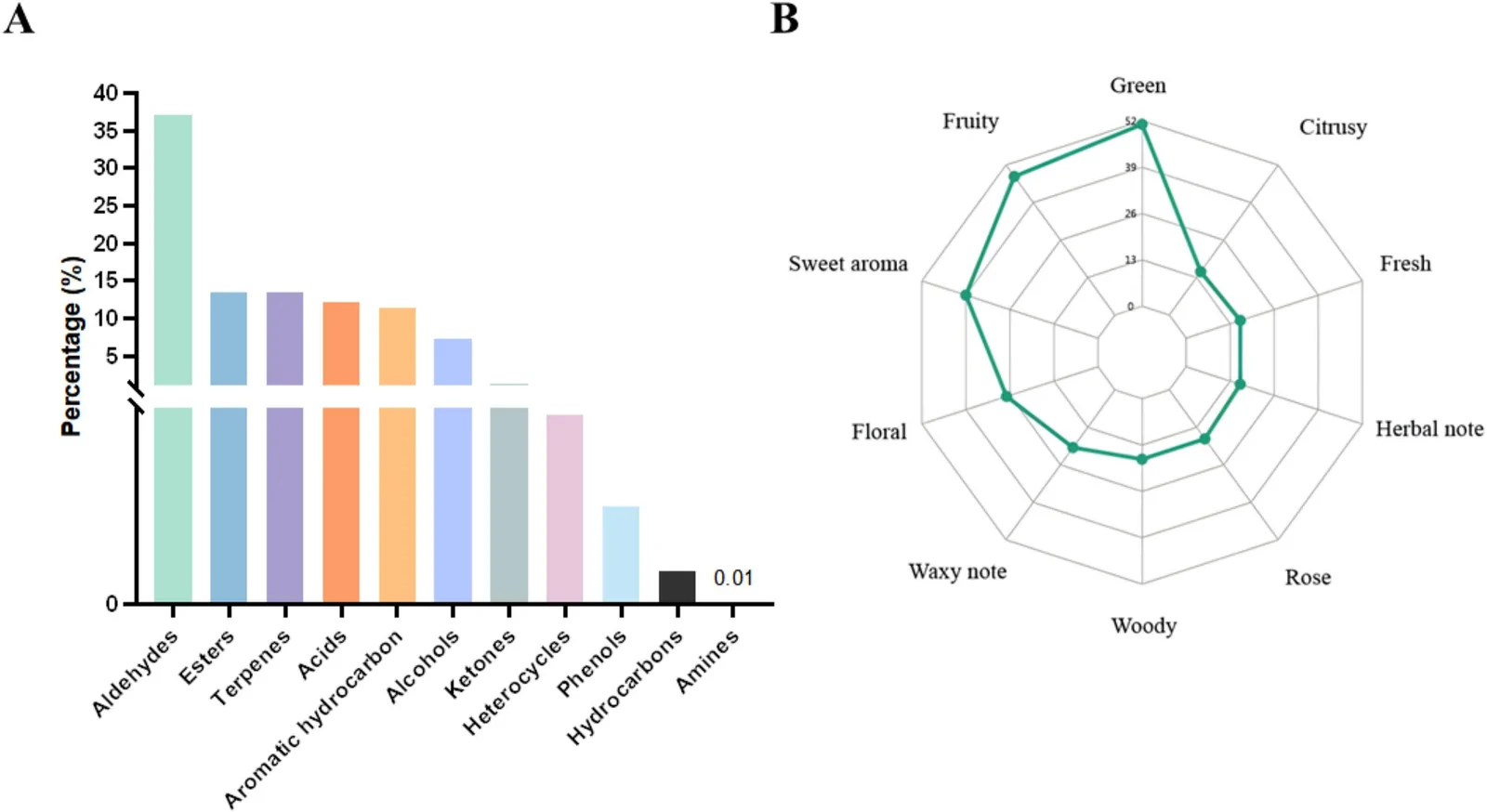
Integrated flavor characterization of garlic paste samples. (A) Category and proportion of aroma components in different garlic paste samples. (B) Radar chart of the major odor descriptors associated with volatile compounds in the tea-treated garlic sample.

The content percentage of each category is shown in Fig. 4A. The total amount of aldehydes was the most, 37.04 μg/mL, and No. 37 undecanal accounted for nearly half of the total amount of aldehydes, followed by esters 13.56 μg/mL and terpenes 13.41 μg/mL, acids 12.19 μg/mL, aromatics 11.51 μg/mL, alcohols 7.23 μg/mL, ketones 1.35 μg/mL, 0.97 μg/mL for heterocycles, and only 1 substance for phenols, hydrocarbons and amines, with contents of 0.50 μg/mL, 0.17 μg/mL and 0.01 μg/mL, respectively. Esters were the most abundant class in terms of the number of identified compounds (40), followed by terpenes (25) and aldehydes (20). Meanwhile, fewer compounds were detected in other classes: alcohols and ketones (10 each), heterocycles (9), aromatics (5), and acids (4).

Based on all the retrieved odor information, the top 10 sensory flavors with the highest number of annotations were counted, i.e., green, fruity, sweet, floral, waxy, woody, rose, herbal, fresh, and citrus (Fig. 4B).Among them, green aroma appeared most frequently with 51 substances, including two volatile garlic components, No. 19 2,4-dimethylthiophene and No. 28 3-ethylene-1,2-dithiohex-4-ene; followed by fruity aroma, which appeared 48 times, with No. 76 2-heptenoic acid the most with 11.901 μg/mL; sweet aroma appeared 39 times, with No. 148 benzyl isobutyrate at a content of 1.801 μg/mL the most; herbs appeared 16 times, with No. 102 cocoa aldehyde containing 5.520 μg/mL the most; of the 27 substances with floral odors, 20 with waxy odors, 17 with woody odors, 17 with rose odors, 16 with fresh odors, and 15 with citrus odors, No. 37 undecylenic aldehyde had the highest content, at 18.261 μg/mL.

Eight compounds were associated with at least five of the top 10 sensory flavors. No. 37 undecylenal was associated with seven sensory flavors, while No. 50 citronellal and No. 83 nerolidol were each associated with six. No. 57 heptyl acetate, No. 82 linalyl acetate, No. 123 n-octanol, No. 136 2-nonanol, and No. 144 linalyl formate were each associated with five sensory flavors. Of these eight substances, undecanal No. 37 has a strong odor, nerolidol No. 83 has a long-lasting odor, and n-octanol No. 123 has a strong, sharp odor and will have a more profound effect on the sensory flavors. In summary, the flavor exhibited by the garlic paste with tea added was mainly green aroma, which was more consistent with the conclusion of sensory evaluation, and the categories that had a greater impact on sensory flavor were aldehydes and esters, with the substances being undecylenic aldehyde, nerolidol, n-octanol, 2-heptenoic acid, benzyl isobutyrate, and cocoa aldehyde. In conclusion, the improved sensory acceptability may result from both the reduction of unpleasant sulfur odors and the masking or modulation effect of tea-derived aroma compounds. GC–MS analysis showed a clear decrease in several sulfur-containing volatile compounds after tea treatment. In addition, 126 aroma-related compounds were detected, many of which were associated with positive aromas such as green, fruity, sweet, and floral notes.

### 3.4 Effects of tea polysaccharides and tea Saponins on DADS clearance

Due to the structural complexity of tea polysaccharides and saponins, their quantitative analysis faces challenges including a lack of standardized methods and limited accuracy. Given these analytical limitations, the contribution of these components was evaluated indirectly by correlating the reduction in their crude content within the tea aqueous extract with the corresponding change in the DADS clearance rate.

The DADS clearance rate of green and dark teas before and after treatment with tea polysaccharide removal or tea saponin removal are shown in Fig. S1. For tea polysaccharides, the DADS clearance rate before and after treatment was 10.88 ± 0.90% and 11.54 ± 0.86% for green tea and 10.96 ± 0.75% and 10.33 ± 0.78% for dark tea, respectively; for the saponins, the DADS clearance rate before and after treatment was 14.52 ± 0.71% and 14.19 ± 0.63% for green tea and 15.05 ± 0.93% and 14.54 ± 0.84% for dark tea, respectively. The significance levels of all four groups were greater than 0.05, indicating that the reduction or absence of tea polysaccharides or tea saponins did not cause significant effects on DADS clearance. Given the structural similarity between allicin and diallyl disulfide (DADS), previous observations on allicin provide relevant mechanistic insight. Consistent results indicate that removal of tea polysaccharides does not lead to a significant change in the extent of allicin reduction, suggesting that polysaccharides are not the primary contributors to the interaction between tea and sulfur-containing odorants (Zhou, 2012). In summary, tea polysaccharides and tea saponins did not significantly contribute to DADS clearance and therefore were not considered in the subsequent screening of tea fractions.

### 3.5 Results of DADS removal tests using different fractions obtained from the initial fractionation of tea components

The initial fractionation of tea components into different fractions is achieved by liquid-liquid extraction, which exploits differences in the partition coefficients of the constituents between two immiscible solvents (Nie et al., 2013). Different fractions contain different major components and different components are in different major fractions, thus eliminating multiple covariance as much as possible.

Table 4 shows the characteristic component contents and DADS clearance rates of different tea fractions obtained from stepwise fractionation of tea aqueous extracts. In green tea, tea polyphenols were mainly enriched in the aqueous layer after n-butanol extraction, with a content of 2763.44 μg/mL. This layer also contained a high level of free amino acids, reaching 1934.25 μg/mL. Caffeine was mainly found in the chloroform layer, with the highest content of 84.18 μg/mL. The total amount of theaflavins was low, with the highest level detected in the ether layer at 10.96 μg/mL. Thearubigins was mainly enriched in the n-butanol layer, reaching 450.27 μg/mL. Theabrownins showed the highest content in the sodium bicarbonate layer, with a value of 417.32 μg/mL. Among these fractions, the aqueous layer after n-butanol extraction showed the highest DADS clearance rate of 24.96%, followed by the aqueous layer after ether extraction, with a clearance rate of 19.39%.

**Table 4:** Characteristic component contents and DADS clearance rates of different fractions from green tea and dark tea extracts.

Number	Fraction	Tea Polyphenols (μg/mL)	Caffeine (μg/mL)	Free Amino Acids (μg/mL)	Theaflavins (μg/mL)	Thearubigins (μg/mL)	Theabrownins (μg/mL)	DADS Clearance Rate (%)
G1	Petroleum ether layer	1.29 ± 0.16f	0.16 ± 0.03d	0.67 ± 0.00d	0.00 ± 0.00f	0.00 ± 0.00e	0.00 ± 0.00d	2.75 ± 0.72f
G2	Chloroform layer	10.32 ± 0.16f	84.18 ± 4.32a	1.57 ± 0.39d	1.26 ± 0.21e	4.39 ± 0.38e	5.71 ± 0.76d	5.12 ± 0.76f
G3	n-Butanol layer	1110.75 ± 5.18b	26.19 ± 0.76b	151.24 ± 1.03b	7.70 ± 1.21c	450.27 ± 13.72a	215.25 ± 3.80c	15.08 ± 2.07c
G4	Aqueous layer after n-butanol extraction	2763.44 ± 18.62a	19.36 ± 0.69c	1934.25 ± 16.94a	9.10 ± 1.21b	133.98 ± 10.07b	237.22 ± 26.36b	24.96 ± 1.82a
G5	Sodium bicarbonate layer	676.34 ± 4.66c	22.09 ± 0.54c	34.11 ± 1.03c	4.20 ± 0.00d	37.34 ± 3.80c	417.32 ± 7.61a	8.16 ± 1.28e
G6	Diethyl ether layer	123.66 ± 2.46e	0.72 ± 0.08d	3.59 ± 0.39d	10.96 ± 0.06a	3.84 ± 0.19e	7.47 ± 1.52d	11.44 ± 1.55d
G7	Aqueous layer after diethyl ether extraction	418.28 ± 1.86d	1.52 ± 0.09d	2.69 ± 0.67d	1.82 ± 0.24e	19.55 ± 1.01d	14.28 ± 0.38d	19.39 ± 2.09b
D1	Petroleum ether layer	0.86 ± 0.09 g	0.12 ± 0.02f	1.35 ± 0.00d	0.21 ± 0.00f	0.66 ± 0.00e	6.59 ± 0.00d	2.34 ± 0.54e
D2	Chloroform layer	9.89 ± 0.09f	59.72 ± 1.25a	1.57 ± 0.39d	2.24 ± 0.12e	3.51 ± 0.38e	6.59 ± 1.32d	4.03 ± 0.68e
D3	n-Butanol layer	793.55 ± 5.82b	20.58 ± 0.76b	41.96 ± 1.03b	5.95 ± 0.61c	552.41 ± 8.29a	329.47 ± 13.18c	10.17 ± 1.91 cd
D4	Aqueous layer after n-butanol extraction	1320.97 ± 1.61a	8.35 ± 0.54d	668.69 ± 7.77a	4.20 ± 0.00d	96.64 ± 3.80b	915.92 ± 19.77a	16.35 ± 2.67a
D5	Sodium bicarbonate layer	177.42 ± 3.23d	12.16 ± 0.45c	13.69 ± 1.03c	15.75 ± 1.82a	27.46 ± 1.90c	459.06 ± 10.07b	7.21 ± 1.70d
D6	Diethyl ether layer	137.10 ± 1.61e	0.55 ± 0.01ef	4.94 ± 0.78d	8.44 ± 0.06b	2.97 ± 0.57e	6.15 ± 0.76d	12.38 ± 2.14bc
D7	Aqueous layer after diethyl ether extraction	284.95 ± 2.46c	1.42 ± 0.08e	4.26 ± 0.39d	2.03 ± 0.12e	17.13 ± 0.00d	12.52 ± 1.14d	14.40 ± 1.72ab

For dark tea, the aqueous layer obtained after n-butanol extraction contained the highest level of tea polyphenols at 1320.97 μg/mL, a value significantly exceeding that of other fractions. This fraction also contained the most abundant free amino acids (668.69 μg/mL), which was likewise significantly higher than in other fractions. Caffeine was the most abundant in the chloroform layer, with the content of 59.72 μg/mL, which was significantly higher than that of other fractions. Thearubigin was mainly found in the n-butanol layer, with the highest content of 552.41 μg/mL, which was significantly higher than that in other fractions.

Unlike green tea, dark tea showed higher levels of tea pigments in the sodium bicarbonate layer and the aqueous layer after n-butanol extraction. The highest contents of theaflavin and theabrownin were 15.75 μg/mL and 915.92 μg/mL, respectively. The aqueous layer after n-butanol extraction showed the highest DADS clearance rate of 16.35%, followed by the aqueous layer after diethyl ether extraction, with a clearance rate of 14.40%. The aqueous layer after n-butanol extraction also had the highest tea polyphenol content among the dark tea fractions. However, the DADS clearance rates of these dark tea fractions were lower than those of the corresponding green tea fractions. Overall, the DADS scavenging ability of dark tea was weaker than that of green tea.

In the aqueous layer after n-butanol extraction, there was a high content of free amino acids, which were slightly close to the tea polyphenols. This was because the free amino acids were almost all in this fraction and had a very low solubility in other organic solvents, while the tea polyphenols were distributed among different fractions and had a high solubility in other organic solvents. The total amount of tea polyphenols in the seven fractions of green tea was 5104.09 μg/mL, and the total amount of free amino acids was 2128.13 μg/mL, while the total amount of tea polyphenols in the seven fractions of dark tea was 2724.73 μg/mL, and the total amount of free amino acids was 736.45 μg/mL. These results show that the content of tea polyphenols was much higher than other ingredients.

**Fig. 5:**
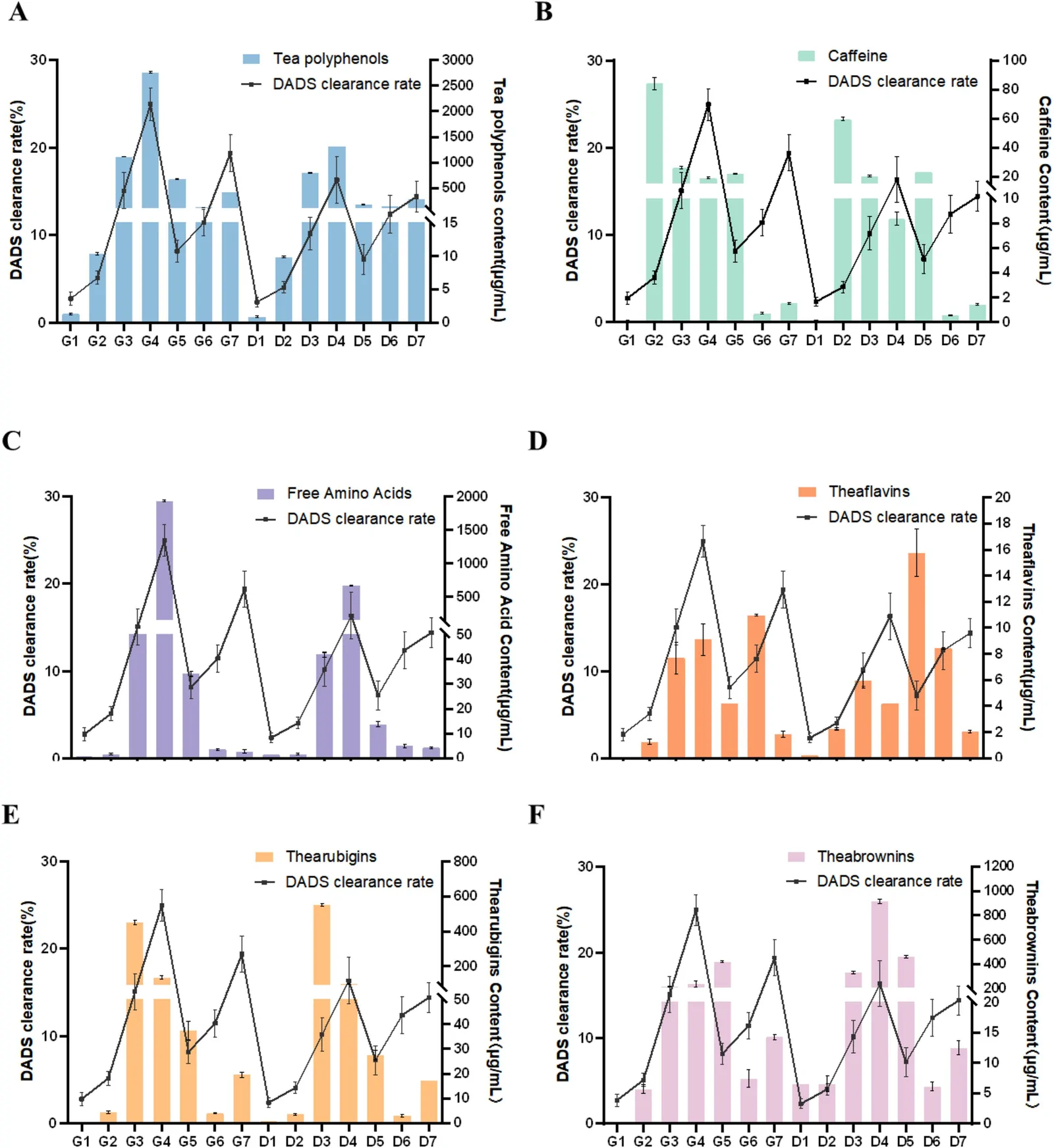
Changes in contents of tea polyphenols (A), caffeine (B), free amino acids (C), theaflavins (D), thearubigins (E), theabrownin (F) and DADS clearance rate.

In addition, operational processes inevitably result in changes in the composition of the tea. First, in order to reduce the impact of organic solvents, the individual fractions were concentrated by rotary evaporation and then redissolved in distilled water, which may have promoted the oxidation of tea polyphenols and increased the tea pigments. Second, emulsification occurred during extraction. Although various emulsion-breaking techniques were adopted, there was still an emulsified layer, so this layer could only be discarded during two-phase separation, which led to different degrees of loss of constituents. The two tea types still differed in the changes in the contents of the characteristic components, and by comparing the trends of these 14 fractions, it can be roughly observed that the trend in tea polyphenol content was more similar to that of DADS clearance than to those of other components, as shown in Fig. 5. However, further analysis is needed to determine the relationship between them.

### 3.6 Screening results of tea characteristic components for DADS clearance

Linear regression analysis was performed based on the data in Table 4. The ANOVA results and regression coefficients are shown in Tables S4 and S5. The regression model was: y = 6.235 + 0.019×₁ – 0.041×₂ – 0.015×₃ + 0.211×₄ – 0.016×₅ – 0.008×₆. In this model, y represents the DADS clearance rate. x₁–x₆ represent tea polyphenol content, caffeine content, free amino acid content, theaflavin content, thearubigin content, and theabrownin content, respectively. The model showed a good fit, with *R* = 0.878, R^2^ = 0.770, and adjusted R^2^ = 0.574. Among these variables, only the coefficient of x₁ was significant, indicating that tea polyphenol content had a significant effect on the DADS clearance rate. The positive coefficient of x₁ also suggested a positive relationship between tea polyphenol content and DADS clearance.

The regression model showed a strong correlation between observed and predicted values (*R* = 0.878). The independent variables together explained 77.0% of the variance in DADS clearance (R^2^ = 0.770), with an adjusted R^2^ of 0.574, indicating that 57.4% of the variance could be reliably attributed to the six variables. The regression model was significant (F = 3.916, *P* = 0.048), demonstrating that the model terms had a statistically meaningful impact on the observed outcome.

After completing the aforementioned multiple linear regression analysis, to optimize the model structure and identify the core variables most explanatory of DADS clearance rates, this study further employed a stepwise regression method for variable screening (Table S6). This approach automatically eliminated insignificant predictor variables, ultimately constructing a more concise and robust predictive model. The new regression model retained only the significant variable, tea polyphenol content (x_1_). The regression model was obtained as follows: y = 7.208 + 0.007 × _1_ (*R* = 0.780, R^2^ = 0.608, adjusted R^2^ = 0.575). Compared to the original full model containing six independent variables (R^2^ = 0.770, adjusted R^2^ = 0.574), the R^2^ value of the new model was expected to decrease, but its adjusted R^2^ demonstrated improved stability. This result indicates that after excluding the interference of insignificant variables such as caffeine, amino acids, and theaflavins, the simplified model based solely on tea polyphenol content can still effectively explain 57.5% of the variation in DADS clearance rate. Furthermore, the model's generalization capability and statistical efficiency may have been optimized.

Several tea components contributed to DADS clearance, with tea polyphenols showing the strongest effect. The content of tea polyphenols in the aqueous layer after ether extraction was not the highest, but tea polyphenols accounted for the highest proportion of the six components in this fraction, reaching 91.30%. This indicates that most impurities were removed and that tea polyphenols were purer in this fraction than in the other fractions. Therefore, the crude tea polyphenol solution was prepared from this fraction.

Tea polyphenols contain numerous substances, among which catechins are the most predominant. Therefore, they were divided into two major categories, catechins and non-catechins, based on the composition of tea polyphenols. As shown in Table S7, after the crude tea polyphenol solution was eluted with different concentrations of methanol, tea polyphenols were mainly detected in the 60% and 70% methanol eluates, while DADS clearance was highest in 70% methanol eluates, followed by 60% methanol eluate, and a highly significant positive linear correlation was observed between tea polyphenol content and DADS clearance. When the methanol concentrations were 0% and 100%, no catechins were detected, but a trace amount of non-catechins still produced a certain removal effect; when the methanol concentrations were 20% and 30%, the contents of non-catechins were almost equal, while the contents of catechins and the removal rate of DADS became significantly larger, indicating the removal effect of catechins. In addition, the tea polyphenol content in the 70% methanol elution was significantly lower than that in the 60% methanol eluate, and the tea polyphenol content in the 80% methanol elution was significantly lower than that in the 50% methanol eluate. However, the DADS clearance rates of the 70% and 80% methanol elution were significantly higher than those of 60% and 50% methanol eluates respectively. At this time the content of catechin analogs was also significantly increased, suggesting that catechin analogs are more effective than non-catechins in DADS clearance. As shown in Table S8, EGCG, ECG, EGC, and EC all significantly reduced DADS compared with the control. The DADS contents after EGCG, ECG, EGC, and EC treatments were 3.38, 3.87, 4.02, and 3.88 μg/g, respectively. No significant differences in DADS content were observed among the four catechin treatments. These results indicate that different catechins may all contribute to DADS reduction. Overall, tea polyphenols showed a strong scavenging effect on DADS and were the main active components in tea responsible for DADS clearance.

The reduction of DADS by tea polyphenols may involve several chemical or physical interactions. Possible mechanisms include oxidation, addition reactions, adsorption, and other non-covalent interactions. However, the exact reaction pathways and products remain unclear. Further studies using advanced analytical methods, such as LC-MS/MS or NMR, are needed to clarify these interactions.

## 4 Conclusions

This study demonstrates that tea can function as a sensory-guided modulator of sulfur-derived garlic odor through the combined effects of sulfur odor reduction and aroma reshaping. Integration of instrumental analysis and sensory evaluation revealed that tea treatment markedly attenuated sulfur-containing odorants responsible for pungent garlic notes, corresponding to an overall reduction of approximately one-third in total sulfur-containing volatiles, and induced a perceptible shift in garlic aroma toward a more balanced and pleasant sensory profile characterized by concurrent tea-derived notes.

Volatile profiling further identified twelve key sulfur-containing compounds as the primary contributors to garlic odor modulation, and comparative treatments indicated that tea was substantially more effective than distilled water in reshaping the sulfur-derived odor profile. Component-level analysis showed that tea polyphenols were the dominant contributors to this modulation, whereas tea polysaccharides and tea saponins exerted no significant effect. Multivariate statistical analysis consistently confirmed the central role of tea polyphenols, yielding a regression model with moderate explanatory power (R^2^ ≈ 0.6) and identifying catechins as particularly influential subcomponents. Collectively, these findings provide quantitative evidence that naturally derived tea components can modulate sulfur-containing garlic odorants and contribute to garlic aroma modulation.

Overall, these findings show that natural tea components can interact with sulfur- containing garlic odorants and provide a food-based strategy for reducing garlic odor. Looking forward, future studies should compare teas from different cultivars, geographical origins, and processing conditions under the same experimental conditions. This will help determine whether the odor-reduction effect can be generalized and support the development of more precise and natural strategies for aroma modulation in complex food systems.

## Ethics and consent to participate declarations

Sensory evaluation was conducted using trained panelists at Sichuan Agricultural University. Formal ethical approval was not required according to institutional guidelines, as the study involved non-invasive food tasting and no identifiable personal data were collected. All participants provided written informed consent prior to participation, and all data were anonymized.

## CRediT authorship contribution statement

**Yongxian Chen:** Writing – review & editing, Writing – original draft, Visualization, Software, Methodology, Investigation, Formal analysis, Data curation, Conceptualization. **Ying Xu:** Writing – review & editing, Writing – original draft, Visualization, Software, Resources, Methodology. **Haibo Zhang:** Writing – review & editing, Visualization, Validation, Software, Methodology. **Hong Huang:** Writing – review & editing, Writing – original draft, Methodology, Investigation, Formal analysis, Data curation. **Ruixin Xue:** Writing – review & editing, Writing – original draft, Visualization, Software. **Xiang Yuan:** Writing – review & editing, Writing – original draft, Validation, Software, Methodology, Investigation, Formal analysis. **Xiangyang Guo:** Writing – review & editing, Writing – original draft, Visualization, Validation, Supervision, Software, Resources, Project administration, Methodology, Investigation, Formal analysis, Data curation, Conceptualization. **Pinwu Li:** Writing – review & editing, Writing – original draft, Visualization, Validation, Supervision, Software, Resources, Project administration, Methodology, Investigation, Funding acquisition, Formal analysis, Data curation, Conceptualization.

## Declaration of competing interest

The authors declare that they have no known competing financial interests or personal relationships that could have appeared to influence the work reported in this paper.

## Data Availability

Data will be made available on request.
