# Supplementary material for: Sensory-guided Design of Garlic Using tea and Identification of key active components
Source: Food Chem X. 2026 Sep 1;39:104379. doi: 10.1016/j.fochx.2026.104379 (PMC13571317; doi:10.1016/j.fochx.2026.104379)
Supplement: Supplementary file 1 [file mmc1.pdf]

Supporting Information

Table S1. Sensory evaluation criteria table.

Garlic Odor Intensity	Score
Negligible garlic odor	8~10
Slight garlic odor	6~8
Moderate garlic odor	4~6
Pronounced irritating garlic odor	2~4
Strong irritating garlic odor	0~2

Table S2. Contents of volatile compounds in garlic paste under untreated group, distilled water treatment, and tea aqueous extract treatment.

Number	Classification	Compounds	Untreated control (μg/g)	Distilled water treatment (μg/g)	Tea aqueous extract treatment (μg/g)
1	Thioethers	Diallyl sulfide	92.87±18.42a	87.01±15.02b	80.70±11.04b
2	Thioethers	Methyl ethyl sulfide	4.16±0.33a	0.71±0.05b	0.43±0.13b
3	Thioethers	Propenyl methyl sulfide	1.67±0.28a	1.58±0.06a	1.66±0.09a
4	Thioethers	Allyl propenyl sulfide	32.02±0.50a	26.87±2.84a	29.66±5.20a
5	Thioethers	Diallyl disulfide	496.04±43.23a	399.93±2.67b	260.84±48.90c
6	Thioethers	Allyl methyl disulfide	77.83±19.12a	28.99±3.54b	27.04±5.23b
7	Thioethers	Propenyl methyl disulfide	3.53±0.83a	4.01±0.32a	3.87±0.30a
8	Thioethers	Allyl propenyl disulfide	194.73±17.65a	152.42±19.46b	131.58±20.34b
9	Thioethers	Isopropyl allyl disulfide	1.81±0.19a	1.32±0.16b	1.24±0.17b
10	Thioethers	Propyl propenyl disulfide	5.33±1.19a	4.65±0.57a	3.61±1.12a
11	Thioethers	Dimethyl trisulfide	5.99±1.48a	2.27±0.70b	1.25±0.23b
12	Thioethers	Diallyl trisulfide	41.670±2.566a	41.21±4.64a	30.92±2.82b

Table S2 (Continued)

Number	Classification	Compounds	Untreated control (μg/g)	Distilled water treatment (μg/g)	Tea aqueous extract treatment (μg/g)
13	Thioethers	Allyl methyl trisulfide	65.42±6.88a	36.52±1.76b	29.93±8.91b
14	Thioethers	Propyl allyl trisulfide	10.52±2.27a	8.98±0.37a	8.00±1.96a
15	Thioethers	1,3-Dipropenyl trisulfide	8.75±1.21a	9.04±0.82a	7.35±2.06a
16	Thioethers	1-Methyl-3-propenyl trisulfide	10.85±0.43a	5.80±0.42b	5.04±0.98b
17	Thioethers	1-Allyl-3-propenyl trisulfide	58.82±5.03a	50.31±3.16ab	42.07±7.83b
18	Heterocyclics	Propylene sulfide	0.03±0.01a	N.D.	N.D.
19	Heterocyclics	2,4-Dimethylthiophene	78.36±11.35a	77.88±8.55a	60.39±13.59a
20	Heterocyclics	3,4-Dimethylthiophene	26.43±1.20b	52.99±4.22a	20.92±3.44b
21	Heterocyclics	1,3-Dithiane	0.06±0.01a	0.04±0.01b	0.04±0.00b
22	Heterocyclics	2-Ethyl-1,3-dithiane	3.93±1.14a	2.64±0.63ab	2.00±0.59b
23	Heterocyclics	1,2-Dithiolane	0.51±0.12b	0.40±0.04b	1.23±0.18a
24	Heterocyclics	1,2-Dithiacyclopentene	162.89±23.88a	145.38±4.08a	85.81±18.01b
25	Heterocyclics	2-Vinyl-1,3-dithiane	30.90±1.90a	18.51±1.42a	17.20±1.16b
26	Heterocyclics	2-Vinyl-4H-1,3-dithiin	24.46±1.357a	17.97±1.66b	14.95±4.08b
27	Heterocyclics	3-Vinyl-4H-1,2-dithiin	1.27±0.147a	0.59±0.16b	0.65±0.01b
28	Heterocyclics	3-Vinyl-1,2-dithiacyclohex-4-ene	234.34±41.92a	123.53±5.45b	111.50±11.31b
29	Heterocyclics	3-Methyl-1,2-dithiacyclopent-3-ene	1.53±0.05a	1.73±0.50a	N.D.
30	Heterocyclics	4-Methyl-1,2,3-trithiacyclopentane	89.53±9.08a	81.25±8.45ab	66.67±13.82b
31	Heterocyclics	1,2,3-Trithiacyclohex-4-ene	97.05±6.22a	78.53±3.35b	57.88±9.27c
32	Heterocyclics	1,3,5-Trithiane	9.45±2.08a	7.44±1.11ab	5.78±1.40b

Table S2 (Continued)

Number	Classification	Compounds	Untreated control (μg/g)	Distilled water treatment (μg/g)	Tea aqueous extract treatment (μg/g)
33	Heterocyclics	5-Methyl-1,2,3,4-tetrathiacyclohexane	82.84±7.15b	84.19±4.82b	114.46±23.04a
34	Thiols	Thiophen-2-ylmethanethiol	N.D.	3.16±0.38a	N.D.
35	Thiols	Allyl mercaptan	N.D.	6.92±0.08a	5.50±0.24b
36	Non－sulfur-containing	Propene	75.06±3.47a	50.42±11.98b	39.84±10.20b

N.D. stands for not detected, the same applies to the following.The letters a, b, c in the table represented significant differences, $p<0.05$.

Table S3. Content and odor of aroma components.

Number	Compounds	Class I	Concentration (μg/mL)	Odor
37	Undecanal	Aldehyde	18.26±1.10	Intense，Waxy note，Soapy scent，Floral fragrance，Citrusy，Green，Fatty odor，Fresh，Rose，Incense-like aroma，Woody，Neroli，Orange peel，Violet floral
38	2-Pentylfuran	Heterocyclic	0.04±0.00	Fruity aroma，Green，Earthy odor，Beany off-flavor，Vegetal note，Metallic tang，Caramel-like aroma，Nutty，Rum，Bready
39	Acetic acid, nonyl ester	Ester	0.37±0.02	Waxy note，Clean，Green，Fruity
40	(2R,5R)-2-methyl-5-(prop-1-en-2-yl)cyclohexanone	Terpenoids	0.69±0.02	Herbal note，Intense aroma
41	β-Selinene	Terpenoids	0.06±0.00	Herbal note

Table S3 (Continued)

Number	Compounds	Class I	Concentration (μg/mL)	Odor
42	(2E,4E)-2,4-Decadienal	Aldehyde	0.02±0.00	Intense aroma，Poultry-like odor，Greasy，Dusty scent，Waxy note，Soapy aroma，Citrusy，Fresh，Orange peel
43	4-(4-methylpent-3-en-1-yl)cyclohex-3-ene-1-carbaldehyde	Aldehyde	0.10±0.00	Citrusy，Greasy，Floral，Marine
44	(Z)-Citral	Terpenoids	0.03±0.00	Sweet aroma，Citrusy lemon，Peel
45	(Z)-Non-3-en-1-ol	Alcohol	0.02±0.00	Fresh，Waxy note，Green，Melon，Peel，Mushroom
46	6-Octenal, 7-methyl-3-methylene-	Aldehyde	0.13±0.01	Lemongrass，Floral，Citrusy lemon，Woody，Rose
47	2-Furanpropanoic acid, ethyl ester	Heterocyclic	0.06±0.00	Tropical pineapple，Fruity，Spicy note，Jammy
48	Propanoic acid, 2-methyl-, 3-phenyl-2-propenyl ester	Ester	0.02±0.00	Sweet aroma，Fruity，Almond，Spicy note
49	Hexanal	Aldehyde	0.02±0.00	Grassy，Green，Leafy，Vinous aroma，Greasy，Apple-like aroma

Table S3 (Continued)

Number	Compounds	Class I	Concentration (μg/mL)	Odor
50	Citronellal	Terpenoids	1.51±0.05	Sweet aroma，Dry，Floral，Herbal note，Waxy note，Citrusy，Grassy，Citrusy lemon，Lemongrass，Rose
51	Myrtenol	Terpenoids	2.35±0.09	Woody，Mint，Grassy，Camphoraceous
52	Ethylene glycol citrate	Terpenoids	3.95±0.25	Cooling，Mint
53	γ-Elemene	Terpenoids	0.05±0.00	Herbal note，Woody
54	Propanoic acid, 2-methyl-, propyl ester	Ester	0.51±0.04	Sweet aroma，Ripe，Fruity，Melon，Berry
55	p-Cresol	Phenol	0.50±0.03	Narcissus floral，Mimosa floral，Smoky，Herbal note
56	2-Nonanone, 3-(hydroxymethyl)-	Ketone	0.21±0.02	Fresh，Sweet aroma，Green，Herbaceous，Earthy odor，Herbal note
57	Acetic acid, heptyl ester	Ester	0.28±0.02	Fruity，Green，Sweet aroma，Herbal note，Pear，Rose，Apricot
58	3-Nonen-1-ol	Alcohol	0.03±0.00	Green，Waxy note，Melon，Cucumber
59	Methyl geranate	Ester	0.82±0.05	Floral，Grassy，Geranium，Citrusy
60	Carvone	Terpenoids	0.06±0.00	Mint，Licorice
61	Salicylic Acid	Acid	0.02±0.00	Subtle，Nutty
62	Benzyl Benzoate	Ester	0.04±0.00	Subtle，Sweet aroma，Balsamic，Greasy，Herbal note，Almond，Plum

Table S3 (Continued)

Number	Compounds	Class I	Concentration (μg/mL)	Odor
63	2-Hexadecanone	Ketone	0.09±0.01	Fruity
64	10-Undecenal	Aldehyde	0.35±0.03	Intense aroma，Coconut，Waxy note，Rose，Citrusy，Soapy aroma， Fatty odor
65	ParAldehyde	Aldehyde	0.12±0.01	Sweet aroma，Aromatic
66	3-Pentanol, 2,3,4-trimethyl-	Alcohol	0.17±0.01	Fruity
67	2-Methyltetrahydrofuran-3-one	Heterocyclic	0.05±0.00	Sweet aroma，Bready note，Buttery，Nutty
68	3-Hexenal	Aldehyde	1.06±0.05	Leafy，Green，Stemmy，Tomato Leaf，Melon，Apple-like aroma
69	2-Hexanoylfuran	Heterocyclic	0.30±0.00	Sweet aroma，Fruity，Green，Apricot，Peach
70	2-Propen-1-ol, 3-phenyl-	Alcohol	0.09±0.01	Sweet aroma，Balsamic，Hyacinth floral，Spicy note，Green，Powdery
71	(+)-Bicyclogermacrene	Terpenoids	0.14±0.00	Green，Woody，Herbaceous

Table S3 (Continued)

Number	Compounds	Class I	Concentration (μg/mL)	Odor
72	Butanoic acid, 3,7-dimethyl-2,6-octadienyl ester, (E)-	Ester	0.03±0.00	Sweet aroma，Fruity，Rose，Waxy note，Raspberry
73	Isobutyl acetate	Ester	0.05±0.00	Sweet aroma，Fruity，Ethereal
74	8-Nonen-2-one	Ketone	0.34±0.02	Fruity，Baked
75	(3E,5E)-Octa-3,5-dien-2-one	Ketone	0.06±0.00	Fruity，Fatty odor，Mushroom
76	2-Heptenoic acid	Acid	11.90±0.76	Green，Grassy，Fruity，Ethereal
77	cis-3-Hexenyl butyrate	Ester	0.06±0.00	Intense aroma，Apple-like aroma，Fresh，Green，Fruity，Vinous aroma，Metallic，Buttery，Creamy
78	Butanoic acid, 1-ethenylhexyl ester	Ester	0.11±0.01	Fruity，Floral，Orchid floral，Waxy note，Green，Ligustrum flower，Metallic，Mushroom，Buttery，Earthy odor
79	p-Menthane-3,8-diol, cis-1,3,trans-1,4-	Terpenoids	2.22±0.12	Herbal note，Eucalyptus，Mint
80	Phenylacetic acid propyl ester	Ester	0.102±0.00	Sweet aroma，Honey，Floral，Apricot，Rose
81	(E)-4-Decenal	Aldehyde	0.54±0.02	Fresh，Citrusy，Orange peel，Green，Fatty odor
82	Linalyl acetate	Terpenoids	0.04 ±0.00	Sweet aroma，Green，Citrusy，Bergamot，Lavender，Woody，Citrusy lemon，Fresh，

Table S3 (Continued)

Number	Compounds	Class I	Concentration (μg/mL)	Odor
83	Nerolidol	Terpenoids	0.30±0.01	Floral，Green，Waxy note，Citrusy，Woody，Rose，Apple-like aroma，Orange Blossom，Lily of the Valley，Long-lasting
84	Acetovanillone	Ketone	0.02±0.00	Subtle，Sweet aroma，Vanilla flavor
85	4-Hexen-3-one	Ketone	0.01±0.00	Pungent，Ethereal，Spicy note，Green，Metallic
86	L-.alpha.-Terpineol	Terpenoids	0.34±0.01	Clove，Floral，Fresh，Lily of the Valley，Subtle
87	Geranyl formate	Ester	1.42±0.08	Fresh，Rose，Orange Blossom，Green，Rose Leaf
88	Cyclopentanone, 2-(2-hexenyl)-	Ketone	0.16±0.01	Greasy，Jasmine sambac，Coconut，Milky，Milky，Floral
89	Benzoic acid, 1-methylethyl ester	Ester	0.44±0.01	Sweet aroma，Fruity，Floral，Balsamic vinegar note
90	n-Butyl laurate	Ester	0.04±0.00	Waxy note，Soapy aroma，Fruity，Roasted peanut
91	Octanoic acid, methyl ester	Ester	0.37 ±0.02	Fruity，Citrusy，Vinous aroma，Orange peel

Table S3 (Continued)

Number	Compounds	Class I	Concentration (μg/mL)	Odor
92	cis-Rose oxide	Terpenoids	0.02±0.00	Sweet aroma，Floral，Rose，Geranium，Powdery，Metallic，Lychee，Leathery，Green
93	Benzenepropanoic acid, methyl ester	Ester	0.06±0.01	Honey，Fruity，Vinous aroma，Balsamic，Floral
94	6-Nonen-1-ol, acetate, (Z)-	Ester	0.06±0.00	Melon，Honeydew Melon，Cantaloupe，Green，Pear，Kiwi，Metallic
95	4-Phenyl-3-buten-2-one	Ketone	0.01±0.00	Sweet aroma，Cinnamon，Balsamic，Rhubarb，Greasy，Floral
96	Undecylenic Acid	Acid	0.04±0.00	Sweet aroma，Woody
97	Pantolactone	Ester	0.41±0.03	Smoky Odor，Baked，Bready note
98	1,1-Dimethoxyheptane	Hydrocarbons	0.18±0.01	Green，Ligustrum flower，Fruity，Orange peel
99	1-Methylindane	Aromatics	0.05±0.00	Floral，Vanilla flavor，Spicy note
100	Benzoic acid, methyl ester	Ester	1.80±0.18	Wintergreen，Almond，Floral，Intense aroma，Cherry，Fruity，Ylang-Ylang，Tuberose
101	Butanoic acid, 3-methyl-, 3-methylbutyl ester	Ester	0.23±0.01	Sweet aroma，Fruity，Green，Ripe，Apple-like aroma，Jammy

Table S3 (Continued)

Number	Compounds	Class I	Concentration (μg/mL)	Odor
102	2-Isopropyl-5-methylhex-2-enal	Aldehyde	5.52±0.34	Herbal note，Lavender，Woody，Green，Blueberry，Tomato Leaf
103	n-Amyl isovalerate	Ester	1.86±0.12	Apple-like aroma，Fresh，Fruity
104	Pulegone	Terpenoids	0.02±0.00	Mint
105	Neroli oxide	Terpenoids	0.33±0.01	Green，Herbaceous，Leathery，Herbal note，Narcissus floral，Celery，Orange Blossom
106	1-Nonanol	Alcohol	0.13±0.00	Fresh，Clean，Fatty odor，Floral，Rose，Orange peel，Dusty scent，Musty，Greasy
107	Levomenthol	Terpenoids	0.09±0.01	Mint
108	1,2-Propanedione, 1-phenyl-	Ketone	0.41±0.01	Plastic-like，Greasy，Honey
109	Acetic acid, octyl ester	Ester	1.50±0.04	Green，Earthy odor，Mushroom，Herbal note，Waxy note
110	Hexanoic acid, butyl ester	Ester	0.01±0.00	Tonka，Tropical pineapple，Berry，Apple-like aroma，Green，Vinous aroma，Waxy note，Soapy aroma

Table S3 (Continued)

Number	Compounds	Class I	Concentration (μg/mL)	Odor
111	2,4-Nonadienal, (E,E)-	Aldehyde	0.13±0.00	Melon，Waxy note，Green，Violet，Leafy，Cucumber，Poultry-like odor，Greasy，Fatty odor，Intense aroma，Floral，Fruity
112	trans-Carveol	Terpenoids	0.35±0.01	Coriander，Spearmint
113	2-Hexenal	Aldehyde	0.15 ±0.01	Sweet aroma，Almond，Fruity，Green，Leafy，Apple-like aroma，Plum，Vegetal
114	Isovaleraldehyde propylene glycol acetal	Heterocyclic	0.07±0.01	Sweet aroma，Ethereal，Fruity，Vinous，Sour，Ripe，Milky，Greasy
115	cis-3-Hexen-1-ol	Alcohol	0.03±0.00	Intense aroma，Fresh，Green，Grassy，Leafy，Vegetal，Herbal note，Greasy
116	4-Heptanone	Ketone	0.03±0.00	Fruity，Cheesy odor，Sweet aroma，Tropical pineapple
117	1-Methylcyclohexanol	Alcohol	0.07±0.01	Aromatic，Mint
118	(E,E)-2,4-Hexadienal	Aldehyde	0.02±0.00	Sweet aroma，Green，Spicy note，Floral，Citrusy

Table S3 (Continued)

Number	Compounds	Class I	Concentration (μg/mL)	Odor
119	Isobutyl butyrate	Ester	0.16±0.01	Sweet aroma，Fruity，Tropical pineapple，Rum，Cherry，Apple-like aroma，Ripe，Fruity，Bubbly，Bubblegum
120	Hexanoic acid, methyl ester	Ester	0.03±0.00	Ethereal，Fruity，Tropical pineapple，Apricot，Strawberry，Fruity，Bacon-like
121	2-Ethoxy-3-methylpyrazine	Heterocyclic	0.01±0.00	Hazelnut，Baked，Almond，Tropical pineapple，Earthy odor
122	BenzAldehyde, 3-methyl-	Aldehyde	0.03±0.00	Sweet aroma，Fruity，Cherry
123	N-Octanol	Alcohol	0.02±0.00	Rich，Citrusy，Rose，Sharp，Waxy note，Greasy，Fruity，Sweet aroma，Grassy，Orange Peel
124	Methyl phenethyl ether	Aromatics	3.32±0.22	Green，Floral，Chrysanthemum floral，Jasmine sambac，Metallic，Rose
125	δ-Hexanolactone	Heterocyclic	0.10±0.01	Greasy，Fruity，Coconut
126	Hexanoic acid, propyl ester	Ester	0.09±0.01	Sweet aroma，Fruity，Tropical pineapple，Green
127	4-Methylbenzyl alcohol	Alcohol	0.59±0.04	Mild，Floral

36 **Table S3** (Continued)

Number	Compounds	Class I	Concentration (μg/mL)	Odor
128	2-Furanacrylaldehyde	Heterocyclic	0.23±0.02	Green，Grassy，Fruity，Spicy note，Vanilla flavor，Nutty，Woody，Cinnamon
129	cis-3-Hexenyl glyceryl acetal	Aldehyde	9.88±0.65	Green，Violet，Green，Leafy，Mushroom，Earthy odor
130	Furan, 3-(4-methyl-3-pentenyl)-	Terpenoids	0.63±0.04	Woody
131	Terpinen-4-ol	Terpenoids	0.05±0.00	Rosin，Peppery，Earthy odor，Woody
132	Isomenthone	Terpenoids	0.07±0.00	Mint，Cooling，PepperyMint，Sweet aroma
133	Lilac Aldehyde D	Aldehyde	0.08±0.00	Sweet aroma，Fresh floral bouquet
134	Isopinocarveol	Terpenoids	0.02±0.00	Woody，Intense aroma，Balsamic
135	Butanoic acid, hexyl ester	Ester	0.01 ±0.00	Intense aroma，Fruity，Green，Sweet aroma，Apple-like aroma，Waxy note，Soapy aroma，Vegetal，Tropical pineapple
136	2-Nonanol	Alcohol	6.09 ±0.40	Rose，Waxy note，Green，Creamy，Citrusy，Orange peel，Cheesy odor，Fruity
137	Dihydrocarveol	Terpenoids	0.02±0.00	Spearmint，Peppery，Spicy note

37 **Table S3** (Continued)

Number	Compounds	Class I	Concentration (μg/mL)	Odor
138	β-Nitroethylbenzene	Aromatics	7.92±0.47	Fresh floral bouquet
139	Carveol	Terpenoids	0.04±0.00	Mint，Spearmint，Cooling，Green，Herbal note，Coriander，Spicy note
140	Hexyl crotonate	Ester	0.35 ±0.01	Sweet aroma，Caramel-like aroma，Green，Walnut，Greasy，Radish，Fruity，Woody，Tropical pineapple
141	cis-7-Decenal	Aldehyde	0.05±0.00	Citrusy，Cucumber
142	Isopentyl hexanoate	Ester	0.03±0.00	Fruity，Apple-like aroma，Tropical pineapple，Green
143	Phenylacetaldehyde dimethyl acetal	Aromatics	0.11±0.00	Green，Leafy，Floral，Rose，Earthy odor，Mushroom
144	1,6-Octadien-3-ol, 3,7-dimethyl-, formate	Ester	0.02±0.00	Citrusy，Herbal note，Bergamot，Lavender，Soapy aroma，Fatty odor，Green，Woody，Fresh，Citrusy lemon
145	L-Perillaldehyde	Aldehyde	0.11±0.01	Fresh，Green，Herbal note，Grassy，Sweet aroma，Mint，Cumin，Cinnamon，Roasted peanut
146	Quinoxaline, 2-methyl-	Heterocyclic	0.10±0.01	Baked，Coffee，Nutty，Fruity

Table S3 (Continued)

Number	Compounds	Class I	Concentration (μg/mL)	Odor
147	Nonanoic acid, ethyl ester	Ester	0.06±0.00	Intense aroma，Grape-like，Fruity，Rose，Waxy note，Rum，Vinous aroma，Greasy，Brandy
148	Benzyl isobutyrate	Ester	1.80±0.11	Jasmine sambac，Greasy，Fruity，Sweet aroma，Rose
149	α-Methylcinnamaldehyde	Aldehyde	0.47±0.03	Sweet aroma，Cinnamon，Spicy note，Cassia
150	Decanoic acid, methyl ester	Ester	0.12±0.01	Greasy，Vinous aroma，Fruity，Floral
151	Glycerol triacetate	Ester	0.01±0.00	Mild，Clean，Fruity
152	Ethyl cyclohexanepropionate	Ester	0.02±0.00	Intense aroma，Fruity，Sweet aroma，Tropical pineapple，Peach，Pear，Floral，Honey
153	Geranic acid	Terpenoids	0.04 ±0.00	Green，Fresh，Greasy，Apple-like aroma
154	Methyl isoeugenol	Aromatics	0.12±0.00	Spicy note，Clove，Floral，Woody，Mild，Sweet aroma
155	n-Heptyl hexanoate	Ester	0.03±0.00	Musty，Green，Fresh，Mallow floral，Leafy
156	Phenoxyethyl isobutyrate	Ester	0.05±0.00	Green，Fruity，Waxy note，Apple-like aroma
157	Veratraldehyde	Aldehyde	0.04±0.00	Sweet aroma，Woody，Vanilla flavor，Heliotrope，Rich

Table S3 (Continued)

Number	Compounds	Class I	Concentration (μg/mL)	Odor
158	cis-3-Hexenyl benzoate	Ester	0.03±0.00	Fresh，Green，Leafy，Floral，Orchid floral，Balsamic vinegar note，Fatty odor
159	Menthyl carboxamide	Amine	0.01±0.00	Mild，Mint
160	Cyclopentaneacetic acid, 3-oxo-2-pentyl-, methyl ester	Ester	0.01±0.00	Floral，Greasy，Jasmine sambac，Green
161	Tetradecanoic Acid	Acid	0.23±0.01	Subtle，Waxy note，Fatty odor，Soapy aroma，Coconut，Milky
162	Octanoic acid, octyl ester	Ester	0.15±0.01	Coconut，Greasy，Fruity

Table S4. Analysis of variance table.

Source of Variation	Sum of Squares (SS)	ddf	Mean Square (MS)	F	P-value
Regression	441.89	6	73.65	3.92	0.05
Residual (Error)	131.64	7	18.81		
Total	573.53	13			

Table S5. Table of coefficients of independent variables.

Independent Variable	Variable Name	Coefficient	Significance (P-value)
x_1	Tea Polyphenol Content	0.02	0.04
x_2	Caffeine Content	-0.04	0.45
x_3	Free Amino Acid Content	-0.02	0.70
x_4	Theaflavin Content	0.21	0.48
x_5	Thearubigin Content	-0.02	0.21
x_6	Theabrownin Content	-0.01	0.21

Table S6. Final Model Coefficients from the Stepwise Regression Analysis.

	B	Standard Error	t	p-value
Constant	7.21	1.45	4.97	0.00
Tea Polyphenols	0.01	0.00	4.31	0.00

Table S7. Composition and DADS clearance rate of different eluents .

Methanol Concentration (%)	Tea Polyphenol Content (μg/mL)	Catechins Content (μg/mL)	Non-catechin Content (μg/mL)	DADS Scavenging Rate (%)
0	1.34±0.19j	0.00±0.00i	1.34	0.27±0.04h
10	60.00±0.16h	10.52±0.08h	49.48	1.47±0.05h
20	61.29±0.16h	12.24±0.37h	49.05	1.37±0.22h
30	86.83±0.19g	36.64±0.84g	50.19	3.14±0.85g
40	268.28±1.86e	93.38±0.81e	174.90	9.44±1.03e
50	567.74±2.79c	173.38±1.54d	394.36	15.07±0.68d
60	859.68±1.61a	483.45±3.90b	376.23	18.54±1.09b
70	796.77±1.61b	585.99±0.63a	210.78	20.67±0.79a
80	418.28±6.11d	321.42±2.36c	96.86	16.39±0.74c
90	105.86±0.34f	88.54±1.78f	17.32	5.63±0.99f
100	30.16±0.16i	0.00±0.00i	30.16	0.85±0.13h

Table S8. GC-MS analysis of sulfur-containing volatile compounds in garlic paste after treatment with different catechins.

Number	substance	Control (μg/g)	EGCG-treated (μg/g)	ECG-treated (μg/g)	EGC-treated (μg/g)	EC-treated (μg/g)
1	Diallyl sulfide	0.40±0.01a	ND	0.31±0.03b	0.25±0.02c	ND
2	Diallyl disulfide	9.80±0.42a	3.38±0.75b	3.87±0.93b	4.02±0.74b	3.88±1.10b
3	Allyl methyl disulfide	1.38±0.18a	0.66±0.10c	1.08±0.08b	0.45±0.04d	0.67±0.06c
4	Methyl 1-propenyl disulfide	0.21±0.01	ND	ND	ND	ND
5	Allyl 1-propenyl disulfide	5.42±1.16a	2.25±0.46b	2.35±0.57b	1.92±0.35b	1.98±0.36b

Table S8 (Continued)

Number	substance	Control (μg/g)	EGCG-treatd (μg/g)	ECG-treatd (μg/g)	EGC-treatd (μg/g)	EC-treated (μg/g)
6	Dimethyl trisulfide	0.23±0.02a	ND	0.12±0.01b	ND	ND
7	Diallyl trisulfide	0.88±0.09	ND	ND	ND	ND
8	Allyl methyl trisulfide	1.87±0.22a	0.96±0.15b	1.02±0.06b	1.01±0.17b	1.00±0.20b
9	2,4-Dimethylthiophene	0.38±0.05a	0.20±0.04c	0.26±0.05bc	0.33±0.08ab	0.30±0.06abc
10	3,4-Dimethylthiophene	0.42±0.08a	0.27±0.05b	0.31±0.07ab	0.24±0.03b	0.35±0.03ab
11	1,2-Dithiacyclopentene	0.25±0.03a	0.25±0.07a	0.31±0.04a	0.27±0.06a	0.29±0.07a
12	3-Vinyl-1,2-dithiacyclohex-4-ene	2.09±0.22a	0.90±0.11b	0.37±0.06c	0.85±0.11b	0.95±0.09b
13	4-Methyl-1,2,3-trithiolane	1.14±0.26a	0.35±0.03b	0.99±0.26a	0.47±0.12b	0.25±0.03b
14	1,2,3-Trithiacyclohex-4-ene	1.07±0.05a	0.35±0.05bc	0.25±0.06c	0.27±0.08c	0.39±0.01b
15	5-Methyl-1,2,3,4-tetrathiane	0.77±0.03a	ND	0.17±0.01c	0.32±0.01b	ND

ND, not detectable.

Fig. S1. Qualitative and quantitative analysis of DADS

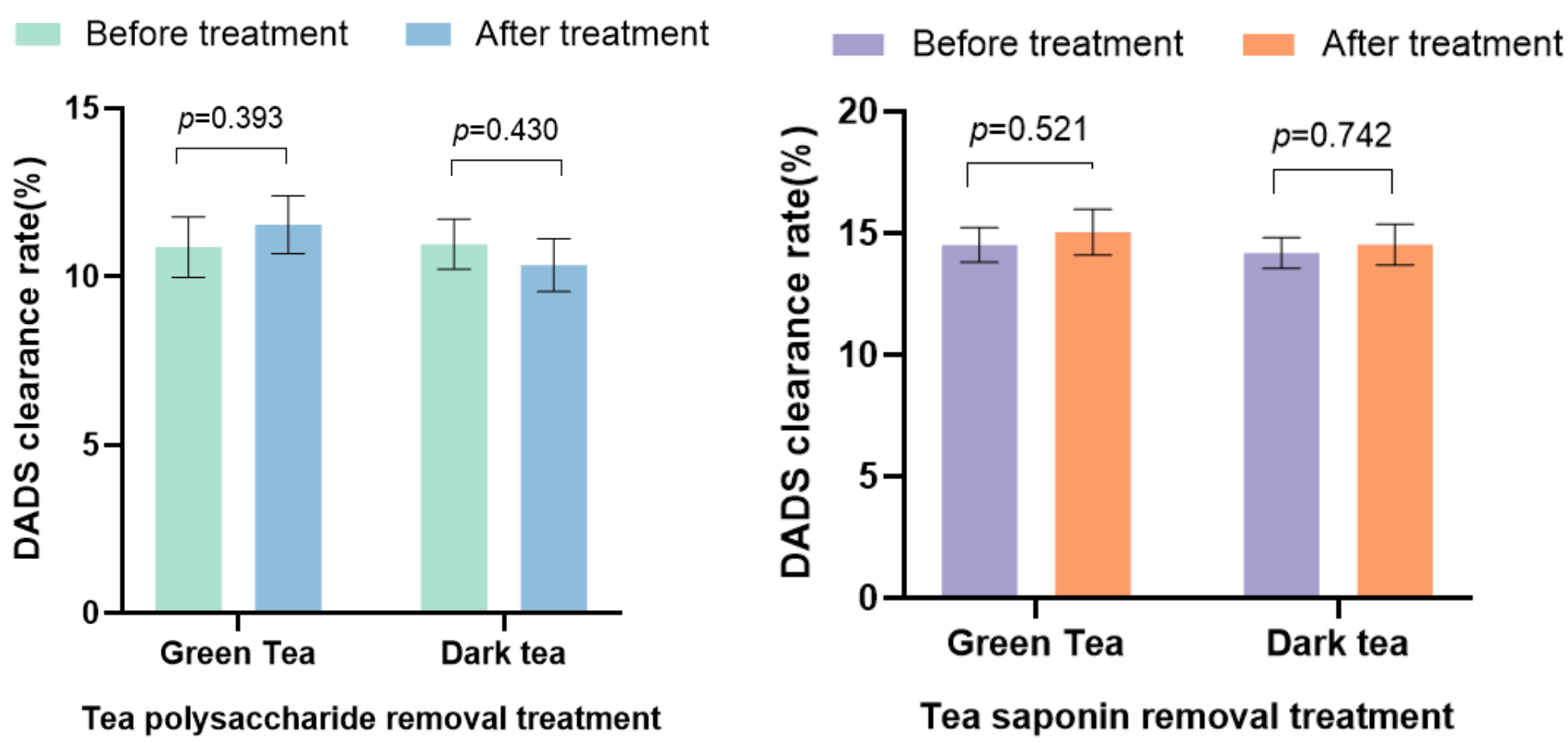

Fig. S2. Qualitative and quantitative analysis of DADS

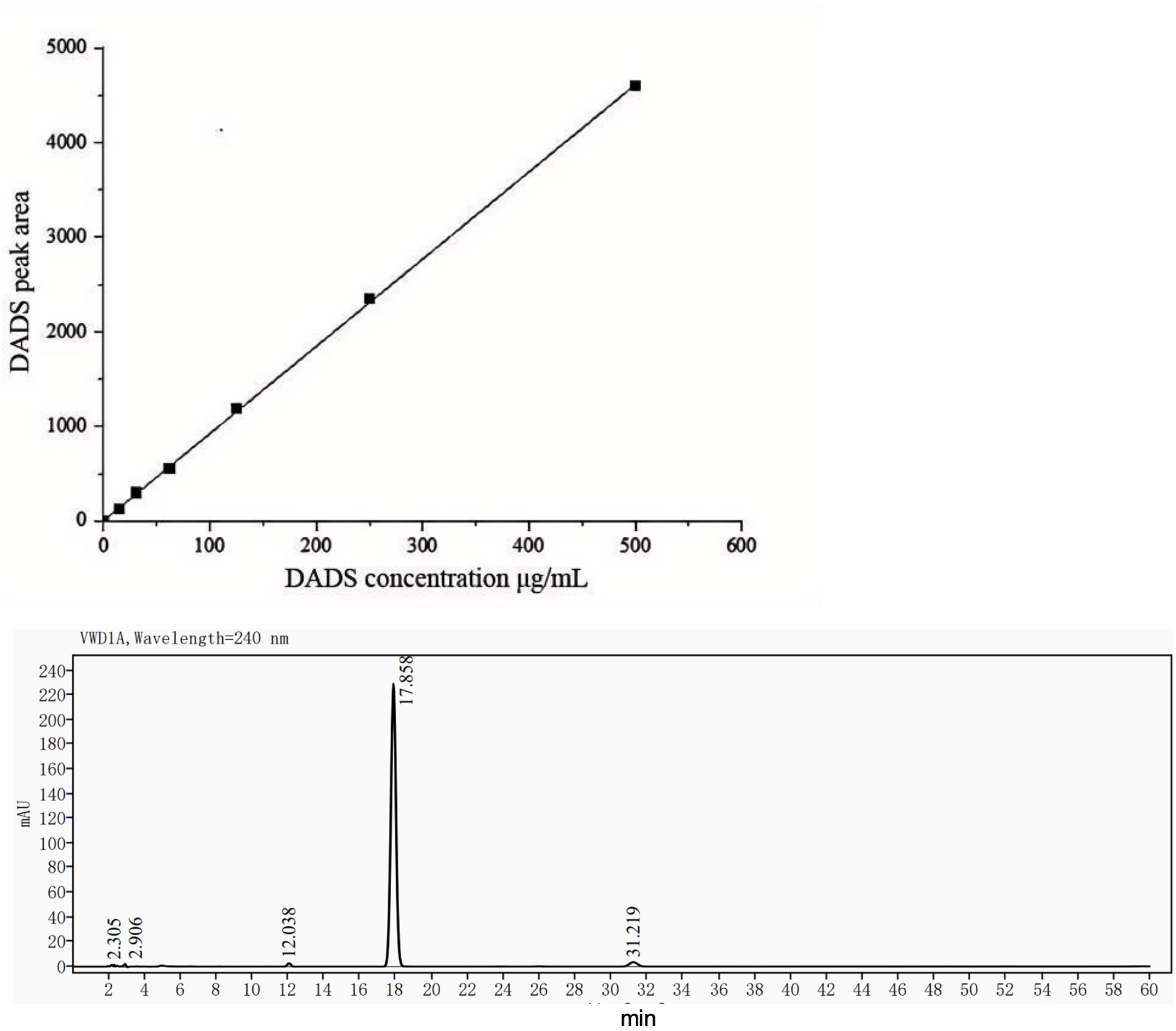

The plot produced by the DADS standard injection, with a distinct peak at 17.858 min, which is the retention time of DADS. Standard curve of DADS with regression equation y = 9.225x + 2.5272 (R^2 = 0.9998), showing excellent linearity up to 500 μg/mL.
